# The type material of Mantodea (praying mantises) deposited in the National Museum of Natural History, Smithsonian Institution, USA

**DOI:** 10.3897/zookeys.433.7054

**Published:** 2014-08-13

**Authors:** Gavin J. Svenson

**Affiliations:** 1 Department of Invertebrate Zoology, Cleveland Museum of Natural History, 1 Wade Oval Drive, Cleveland, Ohio 44106, United States of America

**Keywords:** Taxonomy, Mantodea, praying mantis, holotype, allotype, paratype, lectotype, classification

## Abstract

The collection of Mantodea of the National Museum of Natural History, Smithsonian Institution, includes 26 holotypes, 7 allotypes, 4 lectotypes, 23 paratypes, and 1 paralectotype. Four type specimens were designated as lectotypes within this work. Highly accurate measurement data, high resolution images of specimens and labels, verbatim label data, georeferenced coordinates, original and newly assigned database codes, and bibliographic data are presented for all primary types. Label data for all paratype specimens in the collection are provide in tabular form. The location of the USNM collection has been moved to the Cleveland Museum of Natural History as a loan under the Off-site Enhancement Program.

## Introduction

The collection of Mantodea of the National Museum of Natural History, Smithsonian Institution, includes 26 holotypes, 7 allotypes, 4 lectotypes, 23 paratypes, and 1 paralectotype. The primary type material is mostly from the Afrotropical or Indomalayan regions (62%) while the remaining species are Neotropical. The paratypes largely stem from the revision of *Liturgusa*, which is a Neotropical group (Svenson 2014).

The four species with types considered as syntype material based on the original description were investigated with a literature search. It was found that they lacked a designated holotype or an indication of a sole name-bearing specimen, but rather a group of types, cotypes, or syntypes (see Article 73.2 of the International Code of Zoological Nomenclature, 4^th^ Edition [the Code]). In addition, there was no record of a lectotype designation previous to the current work. Consequently, under Article 74.1 of the Code, lectotypes were designated for *Galapagia solitaria* Scudder, 1893, *Popa batesi* Saussure & Zehntner, 1895, *Stagmomantis hoorie* Caudell, 1910, and *Vates annectens* Rehn, 1900, from syntypes held within the collection of the National Museum of Natural History, Smithsonian Institution to increase taxonomic stability and reduce confusion by creating sole name-bearing specimens for the species above.

In 2012, the collection of Mantodea of the National Museum of Natural History was relocated from Washington, D.C. to the Cleveland Museum of Natural History in Cleveland, Ohio. The collection and the type material are on loan to GJ Svenson under the Off-site Enhancement Program. Svenson oversees curation of the collection and the management of loans related to the collection. All inquiries, loan requests, and visits should be directed to GJ Svenson at the above listed email address.

This paper aims to provide label data, images, georeferenced coordinates, measurements, and references for type material. In addition, this paper aims to resolve confusion regarding the repository of many of these types, which have been incorrectly listed in previous works (see below) as being located in the Academy of Natural Sciences, Philadelphia, the Bernice Pauahi Bishop Museum, Honolulu, the California Academy of Sciences, San Francisco, the Museum of Comparative Zoology, Harvard University, Cambridge, and the Museum für Naturkunde der Humboldt-Universität, Berlin.

## Methods

*Specimen data*: A bibliography for each type was compiled to track use of the species binomen in the taxonomic literature since the original description. The type classification or designation of lectotype is presented followed by all label data directly transcribed using “–” to indicate a line break within a label and “/” to indicate a label break between labels. Codes from the USNM (National Museum of Natural History) were assigned to most of the type material and placed on the specimens, but in some cases these codes are incongruent with those presented in the original descriptions. A possible explanation may be that the codes in the original descriptions were not placed on the specimens. Subsequently, an effort to assign specimen codes across the collection or type collection resulted in the incongruities. Currently, all specimens at the USNM are being labeled with scannable collection codes for databasing, which are reported here as an eight digit numerical code following USNM ENT (National Museum of Natural History, Department of Entomology).

*Georeferencing*: All locality data for type specimens was collected from the primary literature as well as directly transcribed off the labels attached to the specimen. A table is included that contains the type designation, the specimen sex, revised location data that may include additional details not include on the labels sourced from the literature, and georeferenced GPS coordinates in decimal degrees.

*Measurements*: Each type specimen was measured using a Leica M165C stereo-microscope and an IC80 HD coaxial video camera using the live measurements module of the Leica Application Suite (LAS v4.2). Measurements captured in this study are extremely precise using this digital system, which provides a high level of accuracy to users wishing to compare types with other specimens in their study. All measurements presented in this study are in millimeters. A total of 23 measurement classes were captured and defined as:

1. *Body length* = length of body from central ocelli to posterior tip of wing or abdomen (intraspecifically variable measurement, primarily for general size estimation).
2. *Forewing length* = from proximal margin of axillary sclerites to distal tip of the discoidal region.
3. *Hindwing length* = from proximal margin of axillary sclerites to distal tip of the discoidal region.
4. *Pronotum length* = from anterior margin to posterior margin.
5. *Prozone length* = anterior margin of pronotum to center of supra-coxal sulcus.
6. *Pronotum width* = from lateral margins at the widest point including any lamellar expansions, the supra-coxal bulge.
7. *Pronotum narrow width* = from lateral margins of the pronotum at narrowest region of metazone.
8. *Head width* = from lateral margins of the eyes at widest point.
9. *Head vertex to clypeus* = from the vertex of the head at center to the lower margin of the frons and upper margin of clypeus.
10. *Frons width* = from lateral margins of frons, inferior to the antennal insertions, at the widest point.
11. *Frons height* = from upper margin abutting central ocellus to lower margin abutting clypeus.
12. *Prothoracic femur length* = from proximal margin abutting trochanter to distal margin of genicular lobe.
13. *Mesothoracic femur length* = from most proximal margin abutting trochanter to the distal side of the terminal spine insertion site.
14. *Mesothoracic tibia length* = from most proximal groove near joint with the femur to the distal side of the terminal spine insertion site.
15. *Mesothoracic tarsus length* = from proximal joint to the apex of the ungues curve.
16. *Metathoracic femur length* = from most proximal margin abutting trochanter to the distal side of the terminal spine insertion site.
17. *Metathoracic tibia length* = from most proximal groove near joint with the femur to the distal side of the terminal spine insertion site.
18. *Metathoracic tarsus length* = from proximal joint to the apex of the ungues curve.
19. *Discoidal femoral spine count* = all centrally position spines between anteroventral and posteroventral femoral spines (Right/Left).
20. *Anteroventral femoral spine count* = all inner marginal ridge spines and two proximal near marginal spines, but excluding the genicular spine (Right/Left).
21. *Posteroventral femoral spine count* = all outer marginal ridge spines, but excluding the genicular spine (Right/Left).
22. *Anteroventral tibial spine count* = all inner marginal ridge spines, but excluding the distal terminal spur (Right/Left).
23. *Posteroventral tibial spine count* = all outer marginal ridge spines, but excluding the distal terminal spur (Right/Left).

*Imaging*: High resolution images of type specimens were captured using a Passport Storm^©^ system (Visionary Digital™, 2012), which includes a Stackshot z-stepper, a Canon 5D SLR, macro lenses (50mm, 100mm, and MP-E 65mm), three Speedlight 580EX II flash units, and an associated computer running Canon utility and Adobe Lightroom 3.6 software. The z-stepper was controlled through Zerene Stacker 1.04 and images were processed using the P-Max protocol. All images were captured over an 18% grey card background for white balance standards. Images were processed in Adobe Photoshop CS6 Extended to adjust levels, contrast, exposure, sharpness, and add scale bars (10 mm). Minor adjustments were made using the stamp tool to correct background aberrations and to remove distracting debris. Plates were constructed using Adobe Illustrator CS6. Dorsal, ventral and sometimes lateral habitus images were captured depending on specimen mount position and visibility of important features. Images of all labels were captured as well as slide mounted genitalia when available.

Additionally, images were batch processed with Bigshot 2.0 (https://code.google.com/p/bigshot/) to create zoomable image pyramids in HTML5 for deposition on the Project Mantodea server for public access through the project website (found at: http://specimens.mantodearesearch.com under Type specimens).

### Museum codes are as follows

ANSP Academy of Natural Sciences of Drexel University, Philadelphia, PA, USA

BPBM Bernice Pauahi Bishop Museum, Honolulu, Hawai’i, USA

CAS California Academy of Sciences, San Francisco, CA, USA

MCZ Museum of Comparative Zoology, Harvard University, Cambridge, MA, USA

USNM National Museum of Natural History, Smithsonian Institution, Washington, DC, USA

ZMHB Museum für Naturkunde der Humboldt-Universität, Berlin, Germany

## Type material

### Acromantis palauana

Taxon classificationAnimaliaMantodeaHymenopodidae

Beier, 1972

Acromantis palauana : Beier 1972: 174–175; Ehrmann 2002: 53 [Holotype and Allotype listed as deposited in BPBM]; Otte and Spearman 2005: 69.

#### Types.

**Figure 1.**
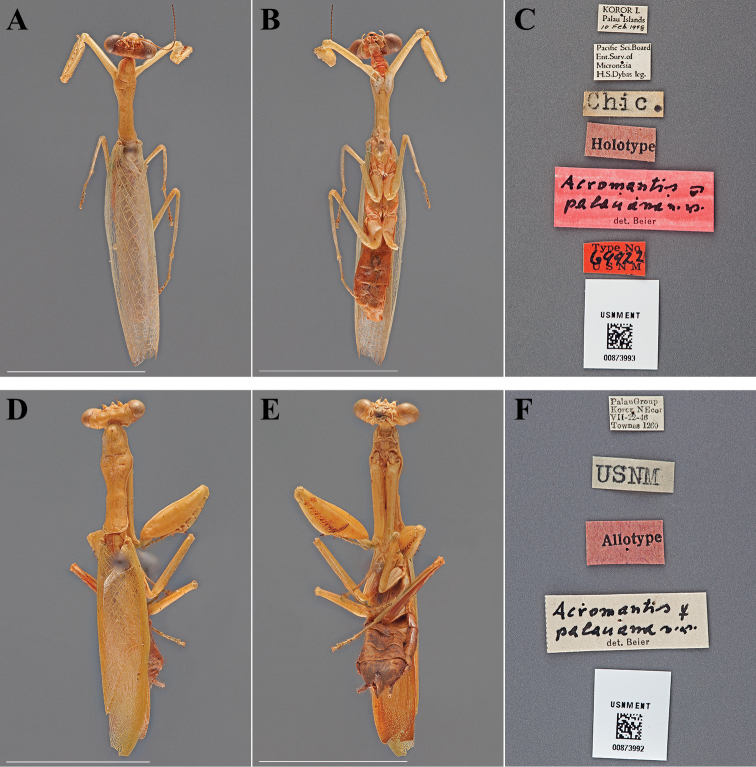
*Acromantis palauana* Beier, 1972 (scale bars = 1 cm). Holotype male (USNM ENT 00873993): **A** dorsal habitus **B** ventral habitus **C** labels. Allotype female (USNM ENT 00873992): **D** dorsal habitus **E** ventral habitus **F** labels.

Holotype Male (Fig. 1A–C; USNM ENT 00873993). Allotype Female (Fig. 1D–F; USNM ENT 00873992).

#### Holotype labels.

Koro I. – Palau Islands – 10 Feb. 1948 / Pacific Sci.Board – Ent. Surv. of – Micronesia – H.S.Dybas leg. / Chic. / Holotype / Acromantis – palauana n.sp. – det. Beier / Type No 69422 USNM.

#### Allotype labels.

Palau Group – Koror NEcor – VII-22-46 – Townes 1260 / USNM / Allotype.

**Table:** 

Holotype	Male	Palau Islands, Koror	10 Feb 1948	7.341023, 134.478913	
Allotype	Female	Palau Islands, Koror	22 Jul 1946	7.341023, 134.478913	

#### Measurements.

*Holotype male*. Body length 23.05; forewing length 15.85; pronotum length 6.05; prozone length 1.80; pronotum width 1.87; pronotum narrow width 1.11; head width 3.83; head vertex to clypeus 1.39; frons width 1.34; frons height 0.42; prothoracic femur length 5.35; mesothoracic femur length 4.22; mesothoracic tibia length 3.25; mesothoracic tarsus length 2.71; metathoracic femur length 4.57; metathoracic tibia length 4.75; metathoracic tarsus length 3.33; discoidal femoral spines R4/L4; anteroventral femoral spine count R13/L13; posteroventral femoral spine count R4/L4; anteroventral tibial spine count R12/L13; posteroventral tibial spine count R12/L11.

*Allotype female*. Body length 24.81; forewing length 14.56; hindwing length 12.46; pronotum length 7.67; prozone length 2.30; pronotum width 2.34; pronotum narrow width 1.41; head width 4.50; head vertex to clypeus 1.79; frons width 1.62; frons height 0.64; prothoracic femur length 6.94; mesothoracic femur length 4.82; mesothoracic tibia length 3.66; mesothoracic tarsus length 2.69; metathoracic femur length 4.14; metathoracic tibia length 5.87; metathoracic tarsus length 3.73; discoidal femoral spines R4/L4; anteroventral femoral spine count R13/L13; posteroventral femoral spine count R4/L4; anteroventral tibial spine count R12/L12; posteroventral tibial spine count R11/L11.

### Ameles malaccana

Taxon classificationAnimaliaMantodeaMantidae

Rehn, 1903

Ameles malaccana : Rehn 1903: 703–704.
Bimantis malaccana : Giglio-Tos 1915: 157; Giglio-Tos 1927: 177; Beier 1935: 30; Ehrmann 2002: 77 [Holotype listed as deposited in ANSP]; Otte and Spearman 2005: 147.

#### Type.

**Figure 2.**
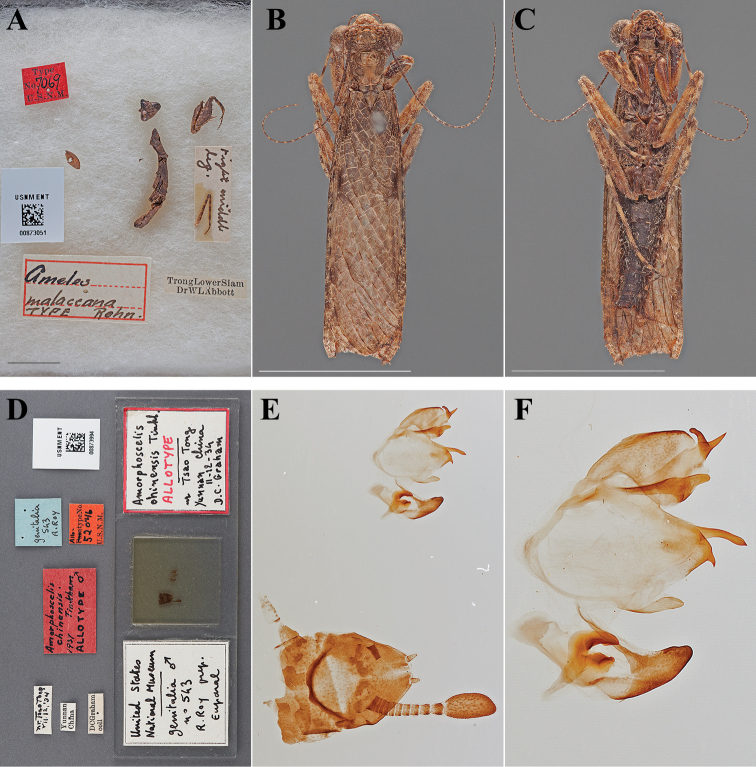
Types (scale bars = 1 cm). *Ameles malaccana* Rehn, 1903 holotype female (USNM ENT 00873051): **A** dorsal habitus and labels in Riker mount. *Amorphoscelis chinensis* Tinkham, 1937 allotype male (USNM ENT 00873994): **B** dorsal habitus **C** ventral habitus **D** labels and genitalic slide mount **E** genital complex and terminal abdominal segments **F** gential complex.

Holotype Female (Fig. 2A; USNM ENT 00873051). The female specimen was referred to as the “Type” by Rehn (1903) and under Article 73.1.1 of the Code this sole name-bearing female specimen is the holotype.

#### Holotype labels.

Trong Lower Siam – Dr WL Abbott / right medial leg. / Ameles – malaccana – TYPE Rehn. / Type – No. 7069 – U.S.N.M. [Cat. No. 6955 USNM; referenced in the original description]

**Table:** 

Holotype	Female	Thailand, Trang	date unknown	7.596958, 99.725938	

#### Measurements.

Body length 22.41; forewing length 4.62; hindwing length 2.54; pronotum length 6.08; prozone length 2.57; pronotum width 3.23; pronotum narrow width 2.21; head width 4.62; head vertex to clypeus 1.69; frons width 1.39; frons height 0.93; prothoracic femur length 6.73; mesothoracic femur length 5.90; mesothoracic tibia length 4.71; mesothoracic tarsus length 3.29; anteroventral femoral spine count R13; posteroventral femoral spine count R4; anteroventral tibial spine count R9; posteroventral tibial spine count R10.

### Amorphoscelis chinensis

Taxon classificationAnimaliaMantodeaAmorphoscelidae

Tinkham, 1937

Amorphoscelis chinensis : Tinkham 1937: 484-485; Roy 1967a: 263; Kaltenbach 1983: 81; Wang and Jin 1995: 197; Yang and Wang 1999: 270; Ehrmann 2002: 61; Otte and Spearman 2005: 22; Roy 2010: 70.

#### Type.

Allotype Male (Fig. 2B–F; USNM ENT 00873994).

#### Allotype labels.

nr. Tsao Tong – VII.12, ‘34 / Yunnan – China / DCGraham – coll / Amorphoscelis – chinensis – 1937 Tinkham – ALLOTYPE ♂ / genitalia – 543 – R.Roy / Allotype No. – 52046 – U.S.N.M.

**Table:** 

Allotype	Male	China, Yunnan, Zhaotong	12 Jul 1934	27.382511, 103.804815	

#### Measurements.

Body length 21.88; forewing length 18.00; pronotum length 2.18; prozone length 0.88; pronotum width 2.64; pronotum narrow width 2.34; head width 4.79; head vertex to clypeus 1.86; frons width 2.04; frons height 0.39; prothoracic femur length 3.63; mesothoracic femur length 5.22; mesothoracic tibia length 3.55; mesothoracic tarsus length 4.39; metathoracic femur length 5.38; metathoracic tibia length 5.79; metathoracic tarsus length 6.41; discoidal femoral spines R1/L1; anteroventral femoral spine count R0/L0; posteroventral femoral spine count R0/L0; anteroventral tibial spine count R0/L0; posteroventral tibial spine count R0/L0.

### Amorphoscelis pantherina

Taxon classificationAnimaliaMantodeaAmorphoscelidae

Roy, 1966

Amorphoscelis pantherina : Roy 1966: 268–270; Kaltenbach 1983: 84; Ehrmann 2002: 62 [Holotype listed as deposited in ANSP]; Otte and Spearman 2005: 25.

#### Type.

**Figure 3.**
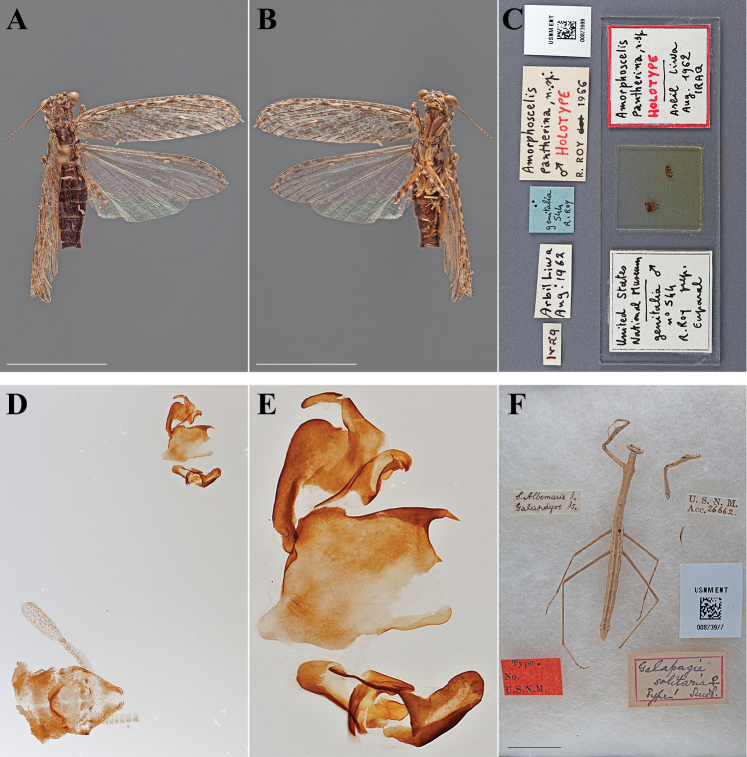
Types (scale bars = 1 cm). *Amorphoscelis pantherina* Roy, 1966 holotype male (USNM ENT 00873999): **A** dorsal habitus **B** ventral habitus **C** labels and genitalic slide mount **D** genital complex and terminal abdominal segments **E** genital complex. *Galapagia solitaria* Scudder, 1893 lectotype female (USNM ENT 00873977): **F** dorsal habitus and labels in Riker mount.

Holotype Male (Fig. 3A–E; USNM ENT 00873999).

#### Holotype labels.

Iraq / Arbil Liwa – Aug. 1962 / genitalia – 544 – R. Roy / Amorphoscelis – pantherina, n. sp. – ♂ HOLOTYPE – R.ROY 1966.

**Table:** 

Holotype	Male	Iraq, Arbil	Aug 1962	36.220434, 43.988001	

#### Measurements.

Body length 20.21; forewing length 16.60; hindwing length 15.25; pronotum length 1.95; prozone length 0.79; pronotum width 2.26; pronotum narrow width 1.93; head width 4.30; head vertex to clypeus 1.71; frons width 1.81; frons height 0.26; prothoracic femur length 3.07; mesothoracic femur length 4.46; mesothoracic tibia length 3.18; mesothoracic tarsus length 3.91; metathoracic femur length 4.62; metathoracic tibia length 4.83; metathoracic tarsus length 4.58; discoidal femoral spines R1/L1; anteroventral femoral spine count R0/L0; posteroventral femoral spine count R0/L0; anteroventral tibial spine count R0/L0; posteroventral tibial spine count R0/L0.

### Calidomantis hosia

Taxon classificationAnimaliaMantodeaMantidae

Rehn, 1912

Calidomantis hosia : Rehn 1912: 464-466.
Miomantis hosia : Giglio-Tos 1927: 369; Beier 1935: 105; Beier 1954: 185; Beier 1969: 32 [Junior SYN of *Calidomantis brunni* Giglio-Tos, 1911]; Ehrmann 2002: 227 [SYN]; Otte and Spearman 2005: 216 [SYN].

#### Type.

**Figure 4.**
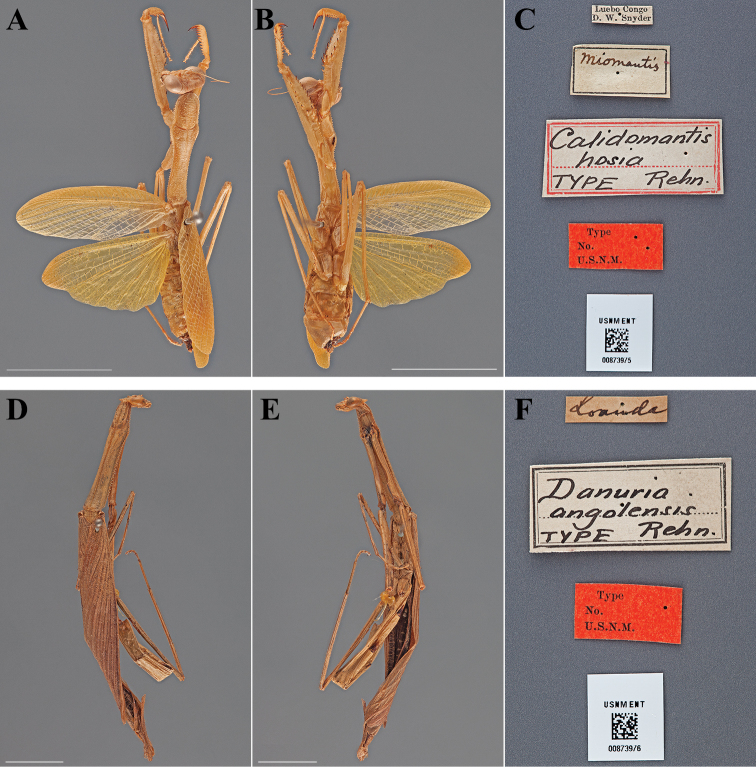
Types (scale bars = 1 cm). *Calidomantis hosia* Rehn, 1912 holotype female (USNM ENT 00873975): **A** dorsal habitus **B** ventral habitus **C** labels. *Danuria angolensis* Rehn, 1912 holotype male (USNM ENT 00873976): **D** dorsal habitus **E** ventral habitus **F** labels.

Holotype Female (Fig. 4A–C; USNM ENT 00873975). The female specimen was referred to as the “Type” by Rehn (1912) and under Article 73.1.1 of the Code this sole name-bearing female specimen is the holotype.

#### Holotype labels.

Luebo Congo – D. W. Snyder / Miomantis / Calidomantis – hosia – TYPE Rehn. / Type – No. – U.S.N.M. [USNM Type No. 14606; referenced in the original description]

**Table:** 

Holotype	Female	Democratic Republic of Congo, Luebo	date unknown	-5.349802, 21.416844	

#### Measurements.

Body length 27.26; forewing length 14.21; hindwing length 12.61; pronotum length 10.78; prozone length 3.23; pronotum width 2.75; pronotum narrow width 1.59; head width 5.86; head vertex to clypeus 2.38; frons width 2.25; frons height 0.83; prothoracic femur length 9.15; mesothoracic femur length 7.89; mesothoracic tibia length 7.23; mesothoracic tarsus length 4.18; metathoracic femur length 10.20; metathoracic tibia length 11.09; metathoracic tarsus length 5.71; discoidal femoral spines R5/L4; anteroventral femoral spine count R13/L13; posteroventral femoral spine count R6 (highly unusual arrangement not including the genicular spine, which suggests a deformation)/L4; anteroventral tibial spine count R11/L11; posteroventral tibial spine count R7/L7. Right forefemur appears to be malformed, which may explain the unusual spine counts.

### Danuria angolensis

Taxon classificationAnimaliaMantodeaMantidae

Rehn, 1912

Danuria angolensis : Rehn 1912: 470–472; La Greca 1954: 286 [Junior SYN of *Danuria barbozae* Bolivar, 1889]; Ehrmann 2002: 115 [SYN]; Otte and Spearman 2005: 303 [SYN].

#### Type.

Holotype Male (Fig. 4D–F; USNM ENT 00873976). The male specimen was referred to as the “Type” by Rehn (1912) and under Article 73.1.1 of the Code this sole name-bearing male specimen is the holotype.

#### Holotype labels.

Loanda / Danuria – angolensis – TYPE Rehn. / Type – No. – U.S.N.M. [USNM Type No. 14609; referenced in the original description]

**Table:** 

Holotype	Male	Angola, Luanda	date unknown	-8.959811, 13.295593	

#### Measurements.

Body length 64.22; forewing length 43.68; pronotum length 19.67; prozone length 5.19; pronotum width 3.86; pronotum narrow width 2.62; head width 5.56; head vertex to clypeus 2.58; frons width 1.94; frons height 0.79; prothoracic femur length 14.79; mesothoracic femur length 13.54; mesothoracic tibia length 11.13; mesothoracic tarsus length 6.76; metathoracic femur length 17.13; metathoracic tibia length 18.01; metathoracic tarsus length 9.46; discoidal femoral spines R4/L4; anteroventral femoral spine count R13/L13; posteroventral femoral spine count R4/L4; anteroventral tibial spine count R11/L11; posteroventral tibial spine count R9/L8.

### Galapagia solitaria

Taxon classificationAnimaliaMantodeaThespidae

Scudder, 1893

Galapagia solitaria : Scudder 1893: 8; Kirby 1904: 276; Hebard 1920b: 317; Giglio-Tos 1927: 221; Beier 1935: 22; Hebard 1935: 280; Terra 1995: 40; Ehrmann 2002: 149 [Holotype and Allotype listed as deposited in MCZ]; Otte and Spearman 2005: 385 [Syntypes listed as deposited in CAS]; Agudelo et al. 2007: 121.

#### Type.

Lectotype Female (Fig. 3F; USNM ENT 00873977). One male and one female were described by Scudder (1893), neither being designated as the sole name-bearing type specimen. Therefore, they are syntypes under Article 72.1.1 of the Code. Herewithin, the female specimen is designated as the lectotype, the male the paralectotype under Article 74.1.1 of the Code. No prior fixation of a sole name-bearing type specimen was found in the literature.

#### Lectotype labels.

S. Albemarle I. – Galapagos Is. / U.S.N.M. – Acc. 26662. / Galapagia – solitaria ♀ – Type! Scudd. / Type. – No. – U.S.N.M.

**Table:** 

Lectotype	Female	Galapagos Islands, South Isabela Island	date unknown	-0.916141, -90.985463	

#### Measurements.

Body length 35.94; pronotum length 9.79; prozone length 3.29; pronotum width 2.29; pronotum narrow width 1.39; head width 3.39; head vertex to clypeus 1.63; frons width 1.19; frons height 0.46; prothoracic femur length 6.24; mesothoracic femur length 7.90; mesothoracic tibia length 7.37; mesothoracic tarsus length 4.47; metathoracic femur length 10.63; metathoracic tibia length 11.12; metathoracic tarsus length 6.94; discoidal femoral spines R4/L4; anteroventral femoral spine count R9/L8; posteroventral femoral spine count R4/L4; anteroventral tibial spine count R5/L6; posteroventral tibial spine count R3/L2.

### Galepsus congicus

Taxon classificationAnimaliaMantodeaTarachodidae

Rehn, 1912

Galepsus congicus : Rehn 1912: 455–457; Giglio-Tos 1927: 102 [Junior SYN of *Galepsus pentheri* Giglio-Tos, 1911]; Ehrmann 2002: 154 [SYN]; Otte and Spearman 2005: 330 [NON-SYN].

#### Type.

**Figure 5.**
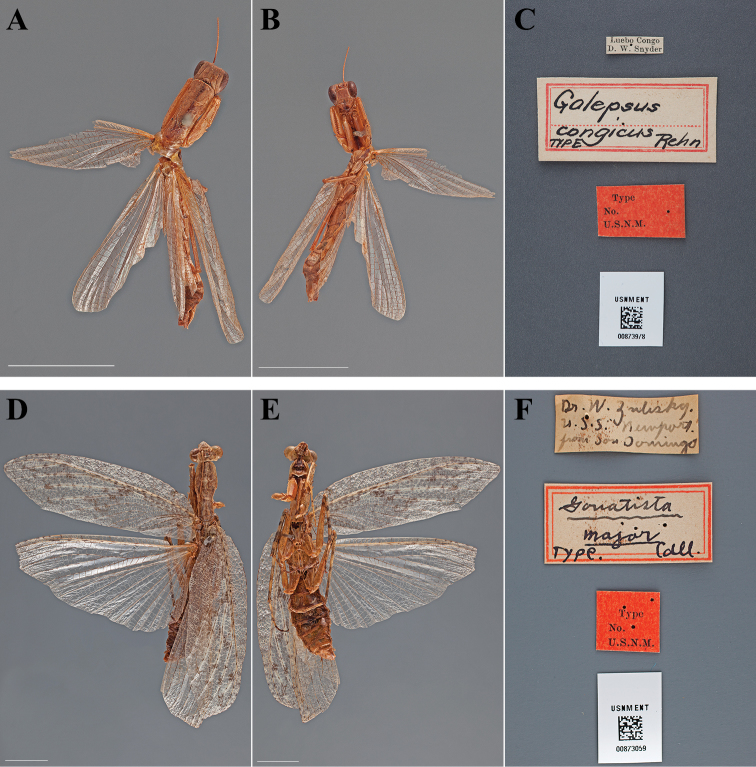
Types (scale bars = 1 cm). *Galepsus congicus* Rehn, 1912 holotype male (USNM ENT 00873978): **A** dorsal habitus **B** ventral habitus **C** labels. *Gonatista major* Caudell, 1912 holotype male (USNM ENT 00873059): **D** dorsal habitus **E** ventral habitus **F** labels.

Holotype Male (Fig. 5A–C; USNM ENT 00873978). The male specimen was referred to as the “Type” by Rehn (1912) and under Article 73.1.1 of the Code this sole name-bearing male specimen is the holotype.

#### Holotype labels.

Luebo Congo – D. W. Snyder / Galepsus – congicus – TYPE Rehn / Type – No. – U.S.N.M. [USNM Type No. 14603; referenced in the original description]

**Table:** 

Holotype	Male	Democratic Republic of Congo, Luebo	date unknown	-5.349802, 21.416844	

#### Measurements.

Body length 28.96; forewing length 19.54; hindwing length 18.80; pronotum length 6.70; prozone length 2.03; pronotum width 2.39; pronotum narrow width 1.98; head width 3.24; head vertex to clypeus 2.28; frons width 1.36; frons height 1.20; prothoracic femur length 5.02; mesothoracic femur length 4.10; mesothoracic tibia length 3.74; mesothoracic tarsus length 2.87; metathoracic femur length 6.19; discoidal femoral spines R4/L4; anteroventral femoral spine count R13/L13; posteroventral femoral spine count R4/L#; anteroventral tibial spine count R11/L12; posteroventral tibial spine count R11/L10.

### Gonatista major

Taxon classificationAnimaliaMantodeaLiturgusidae

Caudell, 1912

Gonatista major : Caudell 1912: 161–162; Giglio-Tos 1927: 290; Beier 1935: 10; La Greca 1940: 306; Terra 1995: 56; Ehrmann 2002: 157; Lombardo and Perez-Gelabert 2004: 35; Otte and Spearman 2005: 128.

#### Type.

Holotype Male (Fig. 5D–F; USNM ENT 00873059).

#### Holotype labels.

Dr. W. Zulisky – U.S.S. Newport – from San Domingo / Gonatista – major – TYPE. Caud. / Type – No. – U.S.N.M. [USNM Cat. No. 15088; referenced in the original description]

**Table:** 

Holotype	Male	Dominican Republic, Santo Domingo	date unknown	18.485982, -69.867603	

#### Measurements.

Body length 63.36; forewing length 49.10; hindwing length 44.93; pronotum length 13.50; prozone length 4.16; pronotum width 5.12; pronotum narrow width 3.50; head width 7.94; head vertex to clypeus 2.97; frons width 2.52; frons height 0.78; prothoracic femur length 13.51; mesothoracic femur length 12.56; mesothoracic tibia length 10.03; mesothoracic tarsus length 7.34; metathoracic femur length 13.83; metathoracic tibia length 16.13; metathoracic tarsus length 9.92; discoidal femoral spines L4; anteroventral femoral spine count L14; posteroventral femoral spine count L4; anteroventral tibial spine count L12; posteroventral tibial spine count L6.

### Harpagonyx carlottae

Taxon classificationAnimaliaMantodeaThespidae

Rehn, 1904

Harpagonyx carlottae : Rehn 1904: 568-569; Kirby 1904: 279; Giglio-Tos 1927: 263 [Junior SYN of *Mionyx saevus* Saussure & Zehntner, 1894]; Terra 1995: 47 [Junior SYN of *Mionyx fera* Saussure & Zehntner, 1894]; Ehrmann 2002: 297 [SYN of *Mionyx fera*];
Pseudomusonia carlottae : Otte and Spearman 2005: 373.

#### Type.

**Figure 6.**
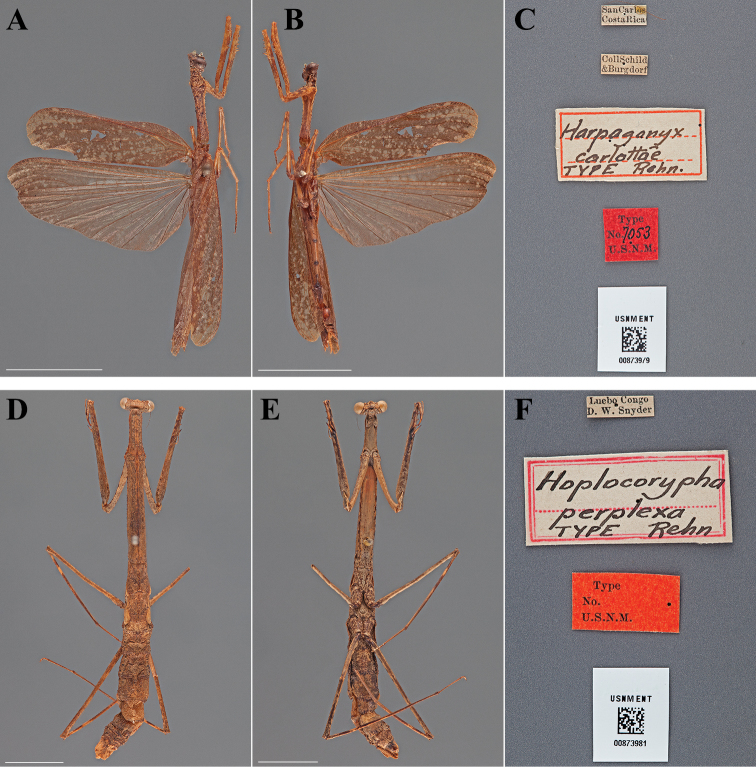
Types (scale bars = 1 cm). *Harpagonyx carlottae* Rehn, 1904 holotype male (USNM ENT 00873979): **A** dorsal habitus **B** ventral habitus **C** labels. *Hoplocorypha perplexa* Rehn, 1912 holotype female (USNM ENT 00873981): **D** dorsal habitus **E** ventral habitus **F** labels.

Holotype Male (Fig. 6A–C; USNM ENT 00873979). The male specimen was referred to as the “Type” by Rehn (1904) and under Article 73.1.1 of the Code this sole name-bearing male specimen is the holotype.

#### Note.

Status uncertain since it has been listed as a junior synonym of *Mionyx saevus* and *Mionyx fera*, both described by Saussure and Zehntner (1894). *Mionyx* is now included within *Pseudomusonia* Werner, 1909. *Mionyx saevus* is currently considered a junior synonym to *Mionyx lineativentris* (Stål, 1877). Otte and Spearman (2005) list H. carlottae as a valid species, but it looks to be mostly accepted as a synonym of *Mionyx fera*.

#### Holotype labels.

San Carlos – Costa Rica / Coll Schild – & Burgdorf / Harpagonyx – carlottae – TYPE Rehn / Type – No. 7053 – U.S.N.M. [USNM Type No. 6953; referenced in the original description]

**Table:** 

Holotype	Male	Costa Rica, San Carlos	date unknown	10.676301, -84.330176	

#### Measurements.

Body length 34.69; forewing length 20.13; hindwing length 19.60; pronotum length 8.74; prozone length 2.26; pronotum width 1.94; pronotum narrow width 1.00; head width 2.80; head vertex to clypeus 1.43; frons width 0.83; frons height 0.37; prothoracic femur length 6.90; mesothoracic femur length 8.03; mesothoracic tibia length 6.74; mesothoracic tarsus length 4.30; metathoracic femur length 9.52; metathoracic tibia length 10.12; metathoracic tarsus length 5.77; discoidal femoral spines R4/L4; anteroventral femoral spine count R10/L10; posteroventral femoral spine count R4/L4; anteroventral tibial spine count L7; posteroventral tibial spine count L4.

### Hoplocorypha boviformis

Taxon classificationAnimaliaMantodeaThespidae

Rehn, 1912

Hoplocorypha boviformis : Rehn 1912: 462–464; Giglio-Tos 1927: 232; Beier 1935: 26; Lombardo 1985: 5; Ehrmann 2002: 186 [Holotype listed as deposited in ANSP]; Otte and Spearman 2005: 361.

#### Type.

**Figure 7.**
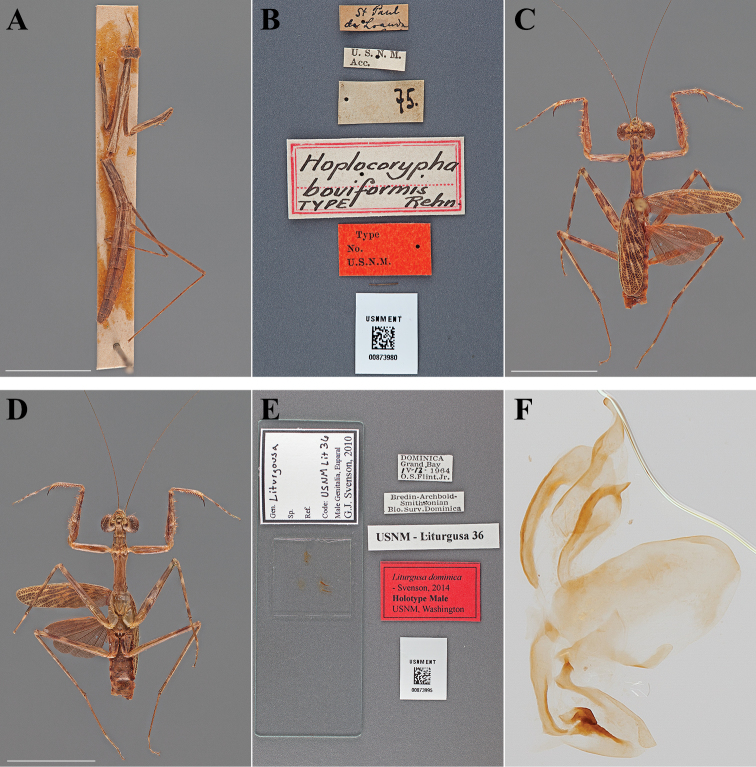
Types (scale bars = 1 cm). *Hoplocorypha boviformis* Rehn, 1912 holotype male (USNM ENT 00873980): **A** dorsal habitus **B** labels. *Liturgusa dominica* Svenson, 2014 holotype male (USNM ENT 00873995): **C** dorsal habitus **D** ventral habitus **E** labels and genitalic slide mount **F** genital complex.

Holotype Male (Fig. 7A–B; USNM ENT 00873980). The male specimen was referred to as the “Type” by Rehn (1912) and under Article 73.1.1 of the Code this sole name-bearing male specimen is the holotype.

#### Holotype labels.

St. Paul – de Loanda / U.S.N.M. – Acc. / 75. / Hoplocorypha – boviformis – TYPE Rehn / Type – No. – U.S.N.M. [USNM Type No. 14605; referenced in the original description]

**Table:** 

Holotype	Male	Angola, Luanda	date unknown	-8.959811, 13.295593	

#### Measurements.

Body length 32.36; pronotum length 11.25; prozone length 3.29; pronotum width 1.70; pronotum narrow width 1.06; head width 3.25; head vertex to clypeus 1.19; frons width 1.07; frons height 0.30; prothoracic femur length 7.64; mesothoracic femur length 8.92; mesothoracic tibia length 7.59; mesothoracic tarsus length 3.96; metathoracic femur length 10.97; metathoracic tibia length 11.82; discoidal femoral spines R4/L4 (small, but distinct spine present proximal to distal 3); anteroventral femoral spine count R12/L12; posteroventral femoral spine count R4/L4; anteroventral tibial spine count R10; posteroventral tibial spine count R4/L4.

### Hoplocorypha perplexa

Taxon classificationAnimaliaMantodeaThespidae

Rehn, 1912

Hoplocorypha perplexa : Rehn 1912: 460–461; Giglio-Tos 1927: 232; Beier 1935: 26; Kaltenbach 1996: 318; Ehrmann 2002: 187 [Holotype listed as deposited in ANSP]; Otte and Spearman 2005: 363.

#### Type.

Holotype Female (Fig. 6D–F; USNM ENT 00873981). The female specimen was referred to as the “Type” by Rehn (1912) and under Article 73.1.1 of the Code this sole name-bearing female specimen is the holotype.

#### Holotype labels.

Luebo Congo – D. W. Snyder / Hoplocorypha – perplexa – TYPE Rehn / Type – No. – U.S.N.M. [USNM Type No. 14604; referenced in the original description]

**Table:** 

Holotype	Female	Democratic Republic of Congo, Luebo	date unknown	-5.349802, 21.416844	

#### Measurements.

Body length 60.73; pronotum length 24.49; prozone length 7.47; pronotum width 3.68; pronotum narrow width 2.87; head width 5.58; head vertex to clypeus 2.62; frons width 2.10; frons height 0.75; prothoracic femur length 16.70; mesothoracic femur length 16.47; mesothoracic tibia length 15.52; mesothoracic tarsus length 7.21; metathoracic femur length 18.88; metathoracic tibia length 19.45; metathoracic tarsus length 7.10; discoidal femoral spines R3/L3; anteroventral femoral spine count R11/L12; posteroventral femoral spine count R4/L4; anteroventral tibial spine count R10/L8; posteroventral tibial spine count R4/L4.

### Liturgusa dominica

Taxon classificationAnimaliaMantodeaLiturgusidae

Svenson, 2014

Liturgusa dominica : Svenson 2014: 110.

#### Types.

**Figure 8.**
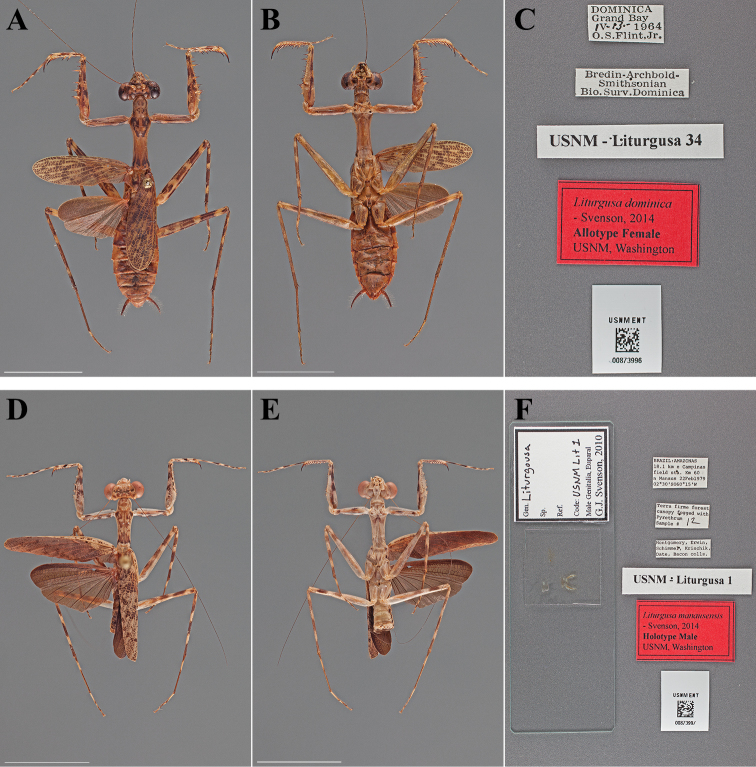
Types (scale bars = 1 cm). *Liturgusa dominica* Svenson, 2014 allotype female (USNM ENT 00873996): **A** dorsal habitus **B** ventral habitus **C** labels. *Liturgusa manausensis* Svenson, 2014 holotype male (USNM ENT 00873997): **D** dorsal habitus **E** ventral habitus **F** labels.

Holotype Male (Fig. 7C–F; USNM ENT 00873995). Allotype Female (Fig. 8A–C; USNM ENT 00873996). 6 Paratypes (USNM ENT 00873019-24).

#### Holotype labels.

DOMINICA – Grand Bay – IV-13-1964 – O.S. Flint, Jr. / Bredin-Archibold- – Smithsonian – Bio. Surv. Dominica / USNM Liturgusa 36 / Liturgusa dominica – Holotype Male USNM – Svenson, 2014.

#### Allotype labels.

DOMINICA – Grand Bay – IV-13-1964 – O.S. Flint, Jr. / Bredin-Archibold- – Smithsonian – Bio. Surv. Dominica / USNM Liturgusa 34 / Liturgusa dominica – Allotype Female USNM – Svenson, 2014.

**Table:** 

Holotype	Male	Dominica, Grand Bay	13 Apr 1964	15.239545, -61.320099	
Allotype	Female	Dominica, Grand Bay	13 Apr 1964	15.239545, -61.320099	
Paratype	Male	Dominica, Grand Bay	13 Apr 1964	15.239545, -61.320099	
Paratype	Female	Dominica, Fond Figues	16 Mar 1964		
Paratype	Male	Dominica, Grand Bay	13 Apr 1964	15.239545, -61.320099	
Paratype	Male	Dominica, Grand Bay	13 Apr 1964	15.239545, -61.320099	
Paratype	Nymph	Dominica, Grand Bay	13 Apr 1964	15.239545, -61.320099	
Paratype	Nymph	Dominica	22–31 Oct 1966		

#### Measurements.

*Holotype male*. Body length 21.18; forewing length 10.74; hindwing length 8.12; pronotum length 6.91; prozone length 2.02; pronotum width 2.01; pronotum narrow width 1.33; head width 4.36; head vertex to clypeus 1.55; frons width 1.51; frons height 0.60; prothoracic femur length 6.02; mesothoracic femur length 8.42; mesothoracic tibia length 6.21; mesothoracic tarsus length 5.83; metathoracic femur length 8.62; metathoracic tibia length 8.70; metathoracic tarsus length 8.57; discoidal femoral spines R4/L4; anteroventral femoral spine count R15/L15; posteroventral femoral spine count R4/L4; anteroventral tibial spine count R10/L10; posteroventral tibial spine count R6/L7.

*Allotype female*. Body length 27.47; forewing length 12.52; hindwing length 9.29; pronotum length 9.04; prozone length 2.66; pronotum width 2.65; pronotum narrow width 1.72; head width 5.22; frons width 2.10; frons height 0.63; prothoracic femur length 7.69; mesothoracic femur length 9.92; mesothoracic tibia length 7.69; mesothoracic tarsus length 6.78; metathoracic femur length 10.01; metathoracic tibia length 10.57; metathoracic tarsus length 9.77; discoidal femoral spines R4/L4; anteroventral femoral spine count R14/L14; posteroventral femoral spine count R4/L4; anteroventral tibial spine count R10/L10; posteroventral tibial spine count R7/L7.

### Liturgusa manausensis

Taxon classificationAnimaliaMantodeaLiturgusidae

Svenson, 2014

Liturgusa manausensis : Svenson 2014: 73.

#### Type.

Holotype Male (Fig. 8D–F; USNM ENT 00873997).

#### Holotype labels.

BRAZIL: AMAZONAS – 18.1 km e Campinas – field sta. Km 60 – n Manaus 22Feb1979 – 02°30'S, 060°15'W / Terra firme forest – canopy fogged with – Pyrethrum – Sample # 12 / Montgomery, Erwin, – Schimmel, Krischik, – Date, Bacon colls. / USNM Liturgusa 1 / Liturgusa manausensis – Holotype Male USNM – Svenson, 2014.

**Table:** 

Holotype	Male	Brazil, Manaus	22 Feb 1979	-2.5000000, -60.250000	

#### Measurements.

Body length 19.40; forewing length 12.93; hindwing length 9.66; pronotum length 5.68; prozone length 1.62; pronotum width 2.07; pronotum narrow width 1.47; head width 4.66; frons width 1.59; frons height 0.54; prothoracic femur length 5.54; mesothoracic femur length 7.27; mesothoracic tibia length 5.72; mesothoracic tarsus length 4.97; metathoracic femur length 7.52; metathoracic tibia length 7.95; metathoracic tarsus length 7.83; discoidal femoral spines R4/L4; anteroventral femoral spine count R15/L15; posteroventral femoral spine count R4/L4; anteroventral tibial spine count R10/L10; posteroventral tibial spine count R7/L7.

### Liturgusa neblina

Taxon classificationAnimaliaMantodeaLiturgusidae

Svenson, 2014

Liturgusa neblina : Svenson 2014: 52.

#### Type.

**Figure 9.**
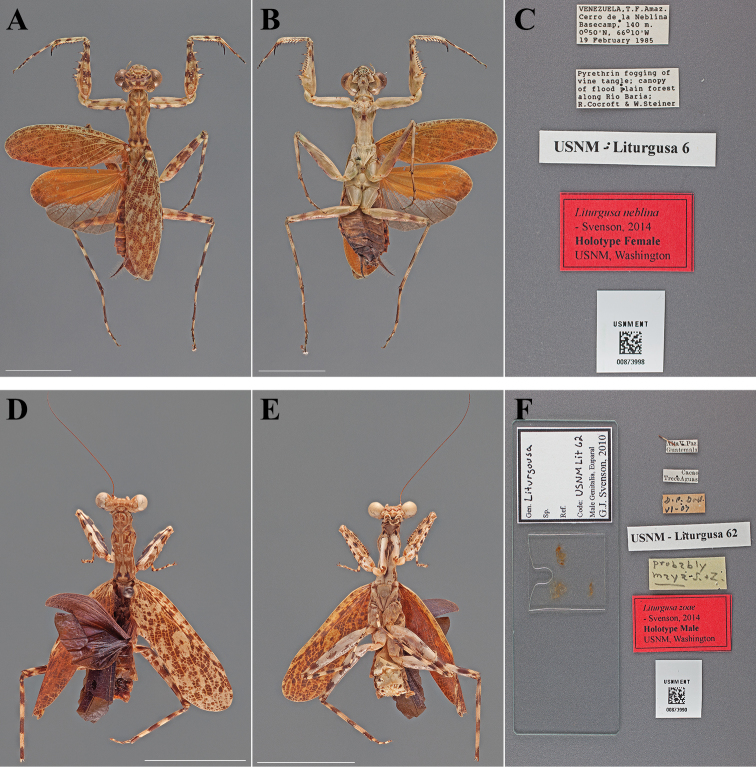
Types (scale bars = 1 cm). *Liturgusa neblina* Svenson, 2014 holotype female (USNM ENT 00873998): **A** dorsal habitus **B** ventral habitus **C** labels. *Liturgusa zoae* Svenson, 2014 holotype male (USNM ENT 00873990): **D** dorsal habitus **E** ventral habitus **F** labels.

Holotype Female (Fig. 9A–C; USNM ENT 00873998).

#### Holotype labels.

VENEZUELA, T.F. Amaz. – Cerro de la Neblina – Basecamp, 140 m. – 0 50'N, 66 10'W – 19 February 1985 / Pyrethrin fogging of – vine tangle; canopy – of flood plain forest – along Rio Baria; – R. Cocroft & W. Steiner / USNM Liturgusa 6 / Liturgusa neblina – Holotype Female USNM – Svenson, 2014.

**Table:** 

Holotype	Female	Venezuela, Cerro de la Neblina	19 Feb 1985	0.833333, -66.166667	

#### Measurements.

Body length 29.75; forewing length 19.54; hindwing length 16.35; pronotum length 8.15; prozone length 2.46; pronotum width 3.68; pronotum narrow width 2.46; head width 6.90; head vertex to clypeus 2.88; frons width 2.78; frons height 1.12; prothoracic femur length 8.45; mesothoracic femur length 9.50; mesothoracic tibia length 7.50; mesothoracic tarsus length 6.48; metathoracic femur length 9.42; metathoracic tibia length 10.72; metathoracic tarsus length 9.13; discoidal femoral spines R4/L4; anteroventral femoral spine count R14/L14; posteroventral femoral spine count R4/L4; anteroventral tibial spine count R10/L10; posteroventral tibial spine count R7/L7.

### Liturgusa zoae

Taxon classificationAnimaliaMantodeaLiturgusidae

Svenson, 2014

Liturgusa zoae : Svenson 2014: 100.

#### Types.

**Figure 10.**
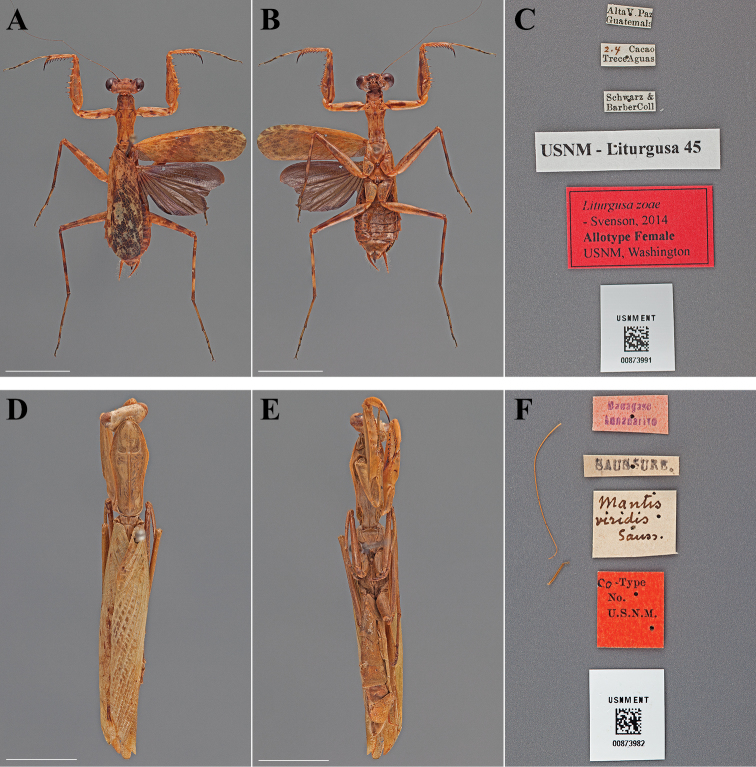
Types (scale bars = 1 cm). *Liturgusa zoae* Svenson, 2014 allotype female (USNM ENT 00873991): **A** dorsal habitus **B** ventral habitus **C** labels. *Mantis viridis* Saussure, 1872 holotype female (USNM ENT 00873982): **D** dorsal habitus **E** ventral habitus **F** labels.

Holotype Male (Fig. 9D–F; USNM ENT 00873990). Allotype Female (Fig. 10A–C; USNM ENT 00873991). Paratype Female (USNM ENT 00873017).

#### Holotype labels.

Alta V. Paz. – Guatemala / Cacao – Trece Aguas / G.P. Goll. – VI - 07 / USNM Liturgusa 62 / Liturgusa zoae – Holotype Male USNM – Svenson, 2014.

#### Allotype labels.

Alta V. Paz. – Guatemala / 2.4 Cacao – Trece Aguas / Schwarz & – Barber Coll. / USNM Liturgusa 45 / Liturgusa zoae – Allotype Female USNM – Svenson, 2014.

**Table:** 

Holotype	Male	Guatemala, Alta V. Paz	Jun 1907	15.592321, -90.146392	
Allotype	Female	Guatemala, Alta V. Paz	Jun 1907	15.592321, -90.146392	
Paratype	Female	Belize, Blue Creek Village	22 Jun 1981	16.195801, -89.043072	

#### Measurements.

*Holotype male*. Body length 23.28; forewing length 15.09; pronotum length 6.61; prozone length 1.89; pronotum width 2.47; pronotum narrow width 1.89; head width 4.95; head vertex to clypeus 1.98; frons width 1.66; frons height 0.66; prothoracic femur length 6.90; mesothoracic femur length 8.61; mesothoracic tibia length 6.49; mesothoracic tarsus length 5.82; metathoracic femur length 8.62; metathoracic tibia length 8.84; discoidal femoral spines R4/L4; anteroventral femoral spine count R14/L15; posteroventral femoral spine count R4/L4; anteroventral tibial spine count R9/L9; posteroventral tibial spine count R7/L7.

*Allotype female*. Body length 27.92; forewing length 17.63; hindwing length 13.94; pronotum length 7.65; prozone length 2.22; pronotum width 3.26; pronotum narrow width 2.44; head width 6.00; head vertex to clypeus 2.44; frons width 2.23; frons height 0.90; prothoracic femur length 7.98; mesothoracic femur length 9.54; mesothoracic tibia length 7.09; mesothoracic tarsus length 6.68; metathoracic femur length 9.15; metathoracic tibia length 9.93; metathoracic tarsus length 9.93; discoidal femoral spines R4/L4; anteroventral femoral spine count R15/L16; posteroventral femoral spine count R4/L4; anteroventral tibial spine count R9/L9; posteroventral tibial spine count R7/L7.

### Mantis viridis

Taxon classificationAnimaliaMantodeaMantidae

Saussure, 1872

Mantis viridis : Saussure 1872: 47–48; Saussure and Zehntner 1895: 201; Giglio-Tos 1912: 19; Rehn 1914: 16; Giglio-Tos 1927: 409; Beier 1935: 92.
Paramantis viridis : Roy 1967b: 144; Kaltenbach 1996: 320; Ehrmann 2002: 265; Otte and Spearman 2005: 266.

#### Type.

Holotype Female (Fig. 10D–F; USNM ENT 00873982). A single female was used in the original description, but not referred to as the type. Under Article 73.1.2 of the Code the female is the holotype by monotypy.

#### Holotype labels.

Madagasc – Annanarivo / SAUSSURE / Mantis – viridis – Sauss / Co-Type – No. – U.S.N.M. [The original publication referenced the following locality: *Habite*: L’Afrique méridionale. Natal.]

**Table:** 

Holotype	Female	Madagascar, Antananarivo	date unknown	-18.921998, 47.495993	

#### Measurements.

Body length 48.57; forewing length 31.78; pronotum length 12.60; prozone length 4.23; pronotum width 5.25; pronotum narrow width 3.55; head width 6.83; head vertex to clypeus 3.28; frons width 2.42; frons height 1.58; prothoracic femur length 12.25; mesothoracic femur length 9.76; mesothoracic tibia length 7.77; mesothoracic tarsus length 6.10; metathoracic femur length 12.53; metathoracic tibia length 12.36; metathoracic tarsus length 8.81; discoidal femoral spines R4/L4; anteroventral femoral spine count R15/L15; posteroventral femoral spine count R4/L4; anteroventral tibial spine count R12/L12; posteroventral tibial spine count R8/L8.

#### Note.

The type status of this specimen is questionable. Saussure did not provide a repository and this specimen is among the type collection of the USNM. However, the locality labels on the specimen do not match the locality mentioned in the original publication. This specimen and the type status needs more investigation.

### Oxyopsis oculea

Taxon classificationAnimaliaMantodeaMantidae

Rehn, 1920

Oxyopsis oculea : Rehn 1920: 231-235; Giglio-Tos 1927: 585; Terra 1995: 66; Ehrmann 2002: 293 [Holotype and Allotype listed as deposited in ANSP]; Otte and Spearman 2005: 293; Agudelo et al. 2007: 124.

#### Types.

**Figure 11.**
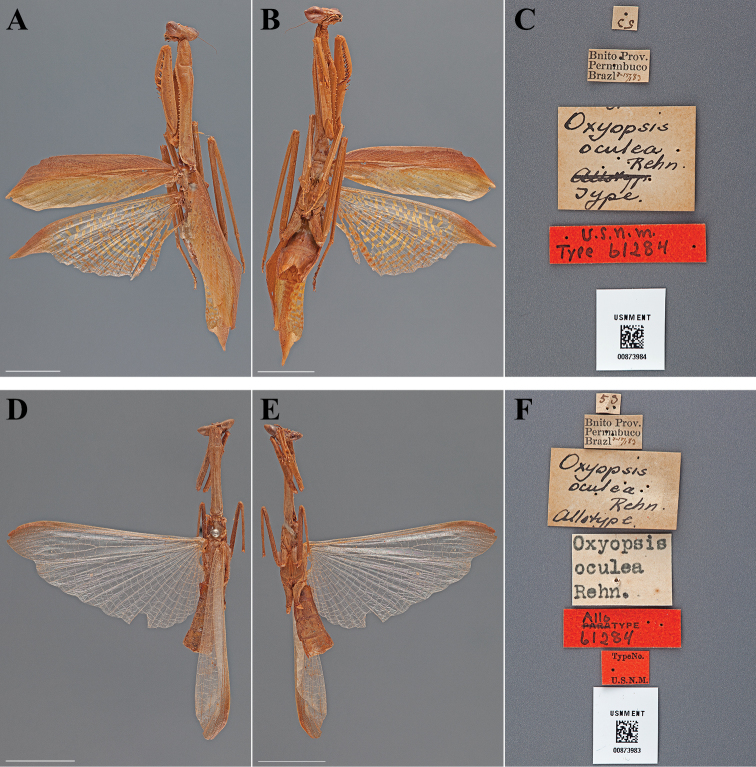
*Oxyopsis oculea* Rehn, 1920 (scale bars = 1 cm). Holotype female (USNM ENT 00873984): **A** dorsal habitus **B** ventral habitus **C** labels. Allotype male (USNM ENT 00873983): **D** dorsal habitus **E** ventral habitus **F** labels.

Holotype Female (Fig. 11A–C; USNM ENT 00873984). Allotype Male (Fig. 11D–F; USNM ENT 00873983).

#### Holotype labels.

53 / Bnito Prov. – Pernmbuco – Brazil 7-15/.82 / Oxyopsis – oculea – Rehn – Type / U.S.N.M. – Type 61284

#### Allotype labels.

53 / Bnito Prov. – Pernmbuco – Brazil 2-15/.82 / Oxyopsis – oculea – Rehn – Allotype / Oxyopsis – oculea – Rehn. / Allotype – 61284 / TypeNo. – U.S.N.M.

**Table:** 

Holotype	Female	Brazil, Pernambuco, Bonito	15 Jul 1883	-8.465728, -35.727823	
Allotype	Male	Brazil, Pernambuco, Bonito	15 Jul 1883	-8.465728, -35.727823	

#### Measurements.

*Holotype female*. Body length 62.70; forewing length 30.57; hindwing length 32.56; pronotum length 23.40; prozone length 5.26; pronotum width 4.31; pronotum narrow width 2.59; head width 8.68; head vertex to clypeus 3.17; frons width 2.83; frons height 1.28; prothoracic femur length 15.56; mesothoracic femur length 13.43; mesothoracic tibia length 12.40; mesothoracic tarsus length 6.73; metathoracic femur length 17.71; metathoracic tibia length 17.99; metathoracic tarsus length 9.13; discoidal femoral spines R4/L4; anteroventral femoral spine count R15/L14; posteroventral femoral spine count R4/L4; anteroventral tibial spine count R#/L#; posteroventral tibial spine count R10/L10.

*Allotype male*. Body length 45.82; hindwing length 28.27; pronotum length 12.44; prozone length 2.90; pronotum width 2.28; pronotum narrow width 1.40; head width 5.80; head vertex to clypeus 1.79; frons width 1.76; frons height 0.60; prothoracic femur length 8.24; mesothoracic femur length 7.94; mesothoracic tibia length 7.09; discoidal femoral spines R4/L4; anteroventral femoral spine count R15/L15; posteroventral femoral spine count R4/L4; anteroventral tibial spine count R17/L16; posteroventral tibial spine count R11/L11.

### Palaeothespis oreophilus

Taxon classificationAnimaliaMantodeaThespidae

Tinkham, 1937

Palaeothespis oreophilus : Tinkham 1937: 498–499; Ehrmann 2002: 381 [Holotype and Allotype listed as deposited in ANSP]; Otte and Spearman 2005: 381.

#### Types.

**Figure 12.**
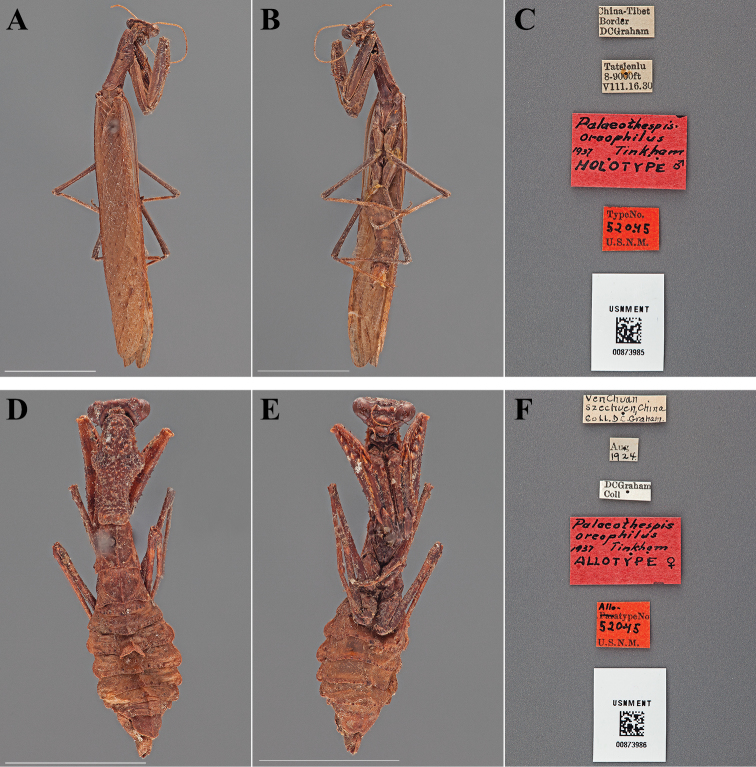
*Palaeothespis oreophilus* Tinkham, 1937 (scale bars = 1 cm). Holotype male (USNM ENT 00873985): **A** dorsal habitus **B** ventral habitus **C** labels. Allotype female (USNM ENT 00873986): **D** dorsal habitus **E** ventral habitus **F** labels.

Holotype Male (Fig. 12A–C; USNM ENT 00873985). Allotype Female (Fig. 12D–F; USNM ENT 00873986).

#### Holotype labels.

China-Tibet – Border – DCGraham / Tatelenlu – 8-9000 ft – VIII.16.30 / Palaeothespis – oreophilus – 1937 Tinkham – HOLOTYPE ♂ / TypeNo. – 52045 – U.S.N.M.

#### Allotype labels.

VenChuan – Szechuen, China – Coll. DC. Graham. / Aug – 1924. / DCGraham – Coll / Palaeothespis – oreophilus – 1937 Tinkham – ALLOTYPE ♀ / Allotype.No. – 52045 – U.S.N.M.

**Table:** 

Holotype	Male	China, Tibet border	16 Aug 1930	30.734037, 98.965915	
Allotype	Female	China, Sichuan, Wenchuan	Aug 1924	31.489086, 103.584293	

#### Measurements.

*Holotype male*. Body length 38.37; forewing length 29.10; pronotum length 6.92; prozone length 2.63; pronotum width 2.92; pronotum narrow width 1.84; head width 4.03; head vertex to clypeus 1.88; frons width 1.51; frons height 0.72; prothoracic femur length 7.57; mesothoracic femur length 7.23; mesothoracic tibia length 6.34; mesothoracic tarsus length 4.80; metathoracic femur length 8.66; metathoracic tibia length 9.17; discoidal femoral spines R4/L4; anteroventral femoral spine count R12/L11; posteroventral femoral spine count R4/L4; anteroventral tibial spine count R9/L8; posteroventral tibial spine count R7/L6.

*Allotype female*. Body length 25.05; pronotum length 7.69; prozone length 2.82; pronotum width 3.74; pronotum narrow width 1.82; head width 4.45; head vertex to clypeus 2.19; frons width 1.71; frons height 0.68; prothoracic femur length 7.74; mesothoracic femur length 7.10; mesothoracic tibia length 6.21; mesothoracic tarsus length 4.35; metathoracic femur length 8.10; metathoracic tibia length 8.37; discoidal femoral spines R4/L4; anteroventral femoral spine count R10/L10; posteroventral femoral spine count R4/L4; anteroventral tibial spine count R8/L8; posteroventral tibial spine count R4/L5.

### Panurgica fratercula

Taxon classificationAnimaliaMantodeaHymenopodidae

Rehn, 1912

Panurgica fratercula : Rehn 1912: 468–469; Giglio-Tos 1927: 553; Beier 1934: 22; Roy 1964: 735; Roy 1965: 577; Ragge and Roy 1967: 586; Gillon and Roy 1968: 1039; Roy and Leston 1975: 297; Ehrmann 2002: 260 [Holotype listed as deposited in ANSP]; Otte and Spearman 2005: 95.

#### Type.

**Figure 13.**
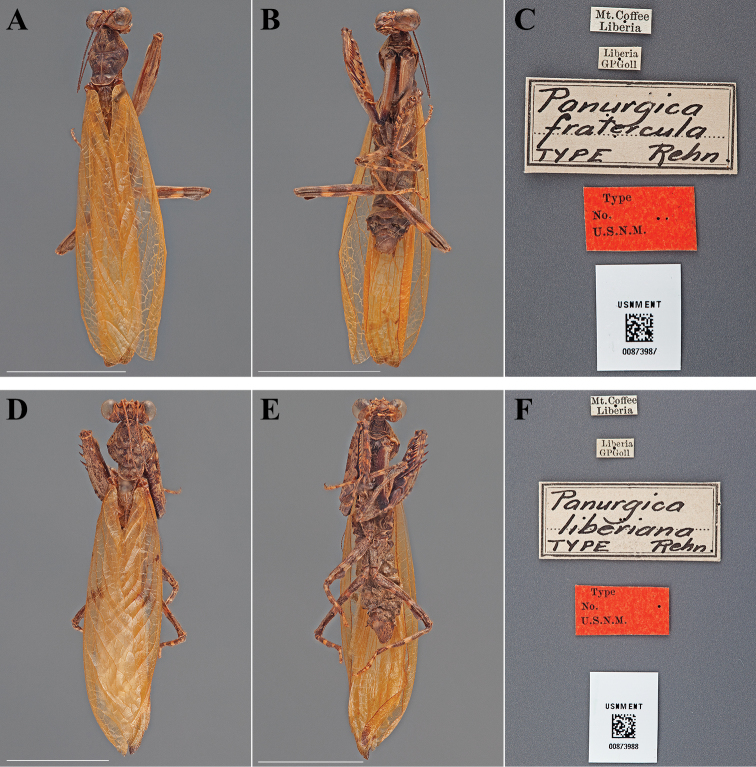
Types (scale bars = 1 cm). *Panurgica fratercula* Rehn, 1912 holotype male (USNM ENT 00873987): **A** dorsal habitus **B** ventral habitus **C** labels. *Panurgica liberiana* Rehn, 1912 holotype male (USNM ENT 00873988): **D** dorsal habitus **E** ventral habitus **F** labels.

Holotype Male (Fig. 13A–C; USNM ENT 00873987). The male specimen was referred to as the “Type” by Rehn (1912) and under Article 73.1.1 of the Code this sole name-bearing male specimen is the holotype.

#### Holotype labels.

Mt. Coffee – Liberia / Liberia – GPGoll / Panurgica – fratercula – TYPE Rehn. / Type – No. – U.S.N.M. [USNM Type No. 14608 reported by Rehn 1912]

**Table:** 

Holotype	Male	Liberia, Mount Coffee	date unknown	6.488618, -10.647978	

#### Measurements.

Body length 28.80; forewing length 22.51; pronotum length 5.16; prozone length 2.38; pronotum width 3.54; pronotum narrow width 1.86; head width 4.69; head vertex to clypeus 2..06; frons width 1.56; frons height 0.67; prothoracic femur length 7.19; mesothoracic femur length 5.78; mesothoracic tibia length 4.48; mesothoracic tarsus length 3.91; metathoracic femur length 6.55; metathoracic tibia length 6.48; metathoracic tarsus length 5.13; discoidal femoral spines R4/L4; anteroventral femoral spine count R12/L12; posteroventral femoral spine count R4/L4; anteroventral tibial spine count R13/L13; posteroventral tibial spine count R13/L13.

### Panurgica liberiana

Taxon classificationAnimaliaMantodeaHymenopodidae

Rehn, 1912

Panurgica liberiana : Rehn 1912: 466-468; Giglio-Tos 1927: 553; Beier 1934: 22; Ehrmann 2002: 260 [Holotype listed as deposited in ANSP]; Otte and Spearman 2005: 95.

#### Type.

Holotype Male (Fig. 13D–F; USNM ENT 00873988). The male specimen was referred to as the “Type” by Rehn (1912) and under Article 73.1.1 of the Code this sole name-bearing male specimen is the holotype.

#### Holotype labels.

Mt. Coffee – Liberia / Liberia – GPGoll / Panurgica – liberiana – TYPE Rehn / Type – No. – U.S.N.M. [USNM Type No. 14607 reported by Rehn 1912]

**Table:** 

Holotype	Male	Liberia, Mount Coffee	date unknown	6.488618, -10.647978	

#### Measurements.

Body length 34.60; forewing length 26.28; hindwing length 24.65; pronotum length 5.77; prozone length 2.69; pronotum width 4.60; pronotum narrow width 2.26; head width 5.28; head vertex to clypeus 2.42; frons width 1.74; frons height 0.96; prothoracic femur length 8.16; mesothoracic femur length 6.18; mesothoracic tibia length 5.34; mesothoracic tarsus length 4.54; metathoracic femur length 6.90; metathoracic tibia length 6.64; metathoracic tarsus length 5.57; discoidal femoral spines R4/L4; anteroventral femoral spine count R12/L12; posteroventral femoral spine count R4/L4; anteroventral tibial spine count R11/L11; posteroventral tibial spine count R12/L12.

### Phyllothelys mitratum

Taxon classificationAnimaliaMantodeaMantidae

Rehn, 1903

Phyllothelys mitratum : Rehn 1903: 715–716; Giglio-Tos 1927: 533; Beier 1935: 128; Ehrmann 2002: 280 [Holotype listed as deposited in ANSP]; Otte and Spearman 2005: 290.

#### Type.

Holotype Female, immature (Fig. 18D; USNM ENT 00873989). The female specimen was referred to as the “Type” by Rehn (1903) and under Article 73.1.1 of the Code this sole name-bearing female specimen is the holotype.

#### Holotype labels.

Trong Lower Siam – Dr WL Abbott / Type – No. 7072 – USNM / Cat. no. – Phyllothelys – mitratum – TYPE Rehn. [Cat. No. 6972, U.S.N.M.; referenced in the original description]

**Table:** 

Holotype	Female	Thailand, Trang	date unknown	7.596958, 99.725938	

#### Measurements.

Body length 27.03; pronotum length 10.15; prozone length 2.29; head width 2.26; head vertex to clypeus 4.41; frons width 0.81; frons height 0.57; prothoracic femur length 6.40; mesothoracic femur length 2.56; mesothoracic tibia length 2.09; mesothoracic tarsus length 2.60; metathoracic femur length 3.38; metathoracic tibia length 3.26; discoidal femoral spines R4/L4; anteroventral femoral spine count R15/L16; posteroventral femoral spine count R4/L4; anteroventral tibial spine count R16/L15; posteroventral tibial spine count R14/L16.

### Popa batesi

Taxon classificationAnimaliaMantodeaMantidae

Saussure & Zehntner, 1895

Popa batesi : Saussure and Zehntner 1895: 230–233; Kirby 1904: 309; Rehn 1911: 26; Giglio-Tos 1927: 631; Paulian 1957: 93; Lombardo 1995: 260 [Junior SYN of *Popa spurca spurca*]; Ehrmann 2002: 287 [SYN]; Otte and Spearman 2005: 306 [NON-SYN].

#### Type.

**Figure 14.**
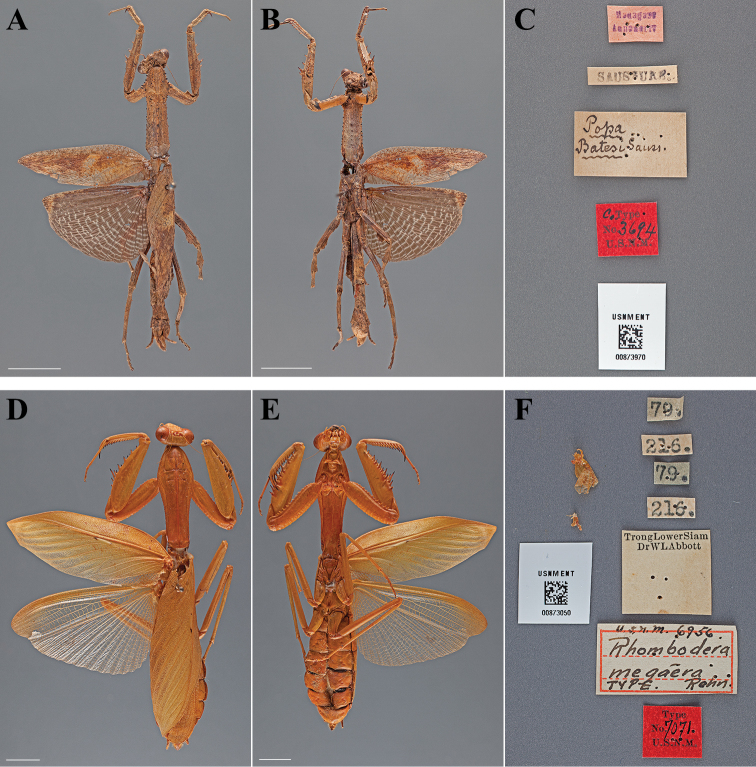
Types (scale bars = 1 cm). *Popa batesi* Saussure & Zehntner, 1895 lectotype female (USNM ENT 00873970): **A** dorsal habitus **B** ventral habitus **C** labels. *Rhombodera megaera* Rehn, 1903 holotype female (USNM ENT 00873050): **D** dorsal habitus **E** ventral habitus **F** labels.

Lectotype Female (Fig. 14A–C; USNM ENT 00873970). One female and one male were described by Saussure and Zehntner (1895), neither being designated as the sole name-bearing type specimen. Therefore, they are syntypes under Article 72.1.1 of the Code. Herewithin, the female specimen is designated as the lectotype, the male the paralectotype under Article 74.1.1 of the Code. No prior fixation of a sole name-bearing type specimen was found in the literature.

#### Lectotype labels.

Madagase – Annanariv / SAUSSURE / Popa – Batesi Sauss. / Co-Type – No. 3694 – U.S.N.M.

**Table:** 

Lectotype	Female	Madagascar, Antanananrivo	date unknown	-18.480912, 47.569213	

#### Measurements.

Body length 56.03; forewing length 24.63; hindwing length 20.51; pronotum length 16.94; prozone length 4.61; pronotum width 5.27; pronotum narrow width 3.49; head width 6.48; head vertex to clypeus 3.18; frons width 2.47; frons height 1.23; prothoracic femur length 11.46; mesothoracic femur length 7.93; mesothoracic tibia length 6.99; mesothoracic tarsus length 6.09; metathoracic femur length 9.77; metathoracic tibia length 10.69; metathoracic tarsus length 6.76; discoidal femoral spines R4/L4; anteroventral femoral spine count R13/L14; posteroventral femoral spine count R4/L4; anteroventral tibial spine count R10/L10; posteroventral tibial spine count R8/L8.

### Pogonogaster latens

Taxon classificationAnimaliaMantodeaThespidae

Hebard, 1919

Pogonogaster latens : Hebard 1919: 136–137; Giglio-Tos 1927: 274; Beier 1935: 16; Terra 1995: 50; Salazar 1999: 10; Ehrmann 2002: 284 [Holotype listed as deposited in ANSP]; Otte and Spearman 2005: 377; Agudelo et al. 2007: 118.

#### Type.

Holotype Female (Fig. 18E; USNM ENT 00873971). The female specimen was referred to as the “Type” by Hebard (1919) and under Article 73.1.1 of the Code this sole name-bearing female specimen is the holotype.

#### Holotype labels.

Rio Aguatal – Colombia. November – 1908. 1800 m. – Coll. A.H. Fasst / Pogonogaster – latens – Hebard – TYPE.

**Table:** 

Holotype	Female	Colombia, Rio Aguacatal	November, 1908	3.454539, -76.547476	

#### Measurements.

Body length 31.57; pronotum length 10.01; prozone length 3.08; prothoracic femur length 8.56; mesothoracic femur length 7.93; mesothoracic tibia length 8.33; mesothoracic tarsus length 6.53; metathoracic femur length 9.27; metathoracic tibia length 9.94; metathoracic tarsus length 8.42; discoidal femoral spines R3/L3; anteroventral femoral spine count R9/L9; posteroventral femoral spine count R4/L4; anteroventral tibial spine count R3/L3; posteroventral tibial spine count R1/L1.

### Rhombodera megaera

Taxon classificationAnimaliaMantodeaMantidae

Rehn, 1903

Rhombodera megaera : Rehn 1903: 710–712; Giglio-Tos 1912: 98; Giglio-Tos 1927: 449; Beier 1935: 85; Ehrmann 2002: 308 [Holotype listed as deposited in ANSP]; Otte and Spearman 2005: 269.

#### Type.

Holotype Female (Fig. 14D–F; USNM ENT 00873050). The female specimen was referred to as the “Type” by Rehn (1903) and under Article 73.1.1 of the Code this sole name-bearing female specimen is the holotype.

#### Holotype labels.

79 / 216 / 79 / 216 / Trong Lower Siam – Dr. WL Abbott / U.S.N.M. 6956 – Rhombodera – megaera – TYPE. Rehn / Type – No. 7071 – U.S.N.M. [Cat. No. 6956, U.S.N.M.; referenced in the original description]

**Table:** 

Holotype	Female	Thailand, Trang	date unknown	7.596958, 99.725938	

#### Measurements.

Body length 94.49; forewing length 53.17; hindwing length 46.19; pronotum length 30.67; prozone length 10.65; pronotum width 10.92; pronotum narrow width 6.84; head width 11.78; head vertex to clypeus 6.35; frons width 4.31; frons height 3.35; prothoracic femur length 23.61; mesothoracic femur length 19.58; mesothoracic tibia length 16.20; mesothoracic tarsus length 9.35; metathoracic femur length 22.03; metathoracic tibia length 22.98; metathoracic tarsus length 12.67; anteroventral femoral spine count R13/L15; posteroventral femoral spine count R4/L4; anteroventral tibial spine count R14/L14; posteroventral tibial spine count R12/L11.

### Stagmatoptera insatiabilis

Taxon classificationAnimaliaMantodeaMantidae

Rehn, 1904

Stagmatoptera insatiabilis : Rehn 1904: 572–573; Kirby 1904: 301; Rehn 1911: 13; Giglio-Tos 1914: 36; Hebard 1923: 340 [Junior synonym of *Stagmomantis theophila* Rehn, 1904]; Giglio-Tos 1927: 384 [SYN]; Hebard 1933: 29 [SYN]; Beier 1935: 96 [SYN]; Terra 1995: 70 [SYN]; Ehrmann 2002: 333 [SYN]; Otte and Spearman 2005: 212 [SYN]; Agudelo et al. 2007: 150 [SYN].

#### Type.

**Figure 15.**
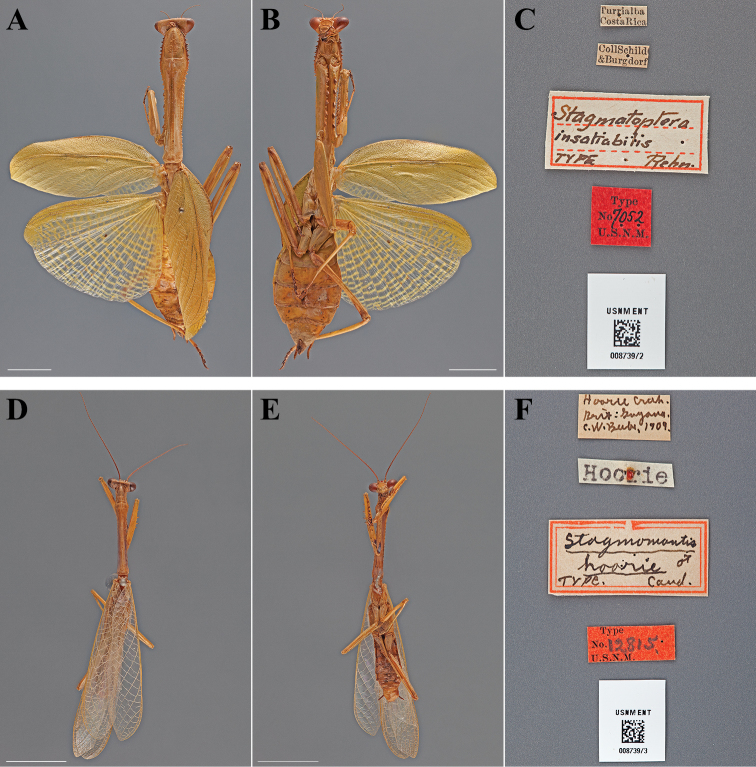
Types (scale bars = 1 cm). *Stagmatoptera insatiabilis* Rehn, 1904 holotype female (USNM ENT 00873972): **A** dorsal habitus **B** ventral habitus **C** labels. *Stagmomantis hoorie* Caudell, 1910 lectotype male (USNM ENT 00873973): **D** dorsal habitus **E** ventral habitus **F** labels.

Holotype Female (Fig. 15A–C; USNM ENT 00873972). The female specimen was referred to as the “Type” by Hebard (1904) and under Article 73.1.1 of the Code this sole name-bearing female specimen is the holotype.

#### Holotype labels.

Turrialba – Costa Rica / Coll Schild – & Burgdorf / Stagmatoptera – insatiabilis – TYPE Rehn. / Type – No. 7052 – U.S.N.M. [Cat. No. 6954, U.S.N.M.; referenced in the original description]

**Table:** 

Holotype	Female	Costa Rica, Turrialba	date unknown	9.904942, -83.688056	

#### Measurements.

Body length 71.73; forewing length 34.77; hindwing length 31.46; pronotum length 29.17; prozone length 6.77; pronotum width 6.20; pronotum narrow width 3.39; head width 8.95; head vertex to clypeus 3.71; frons width 3.13; frons height 1.78; prothoracic femur length 19.35; mesothoracic femur length 16.69; mesothoracic tibia length 13.76; mesothoracic tarsus length 7.37; metathoracic femur length 20.45; metathoracic tibia length 19.51; metathoracic tarsus length 10.47; discoidal femoral spines R4/L4; anteroventral femoral spine count R15/L16; posteroventral femoral spine count R4/L4; anteroventral tibial spine count R13/L13; posteroventral tibial spine count R11/L12.

### Stagmomantis hoorie

Taxon classificationAnimaliaMantodeaMantidae

Caudell, 1910

Stagmomantis hoorie : Caudell 1910: 123–124.
Parastagmatoptera hoorie : Giglio-Tos 1927: 590; Terra 1995: 65; Ehrmann 2002: 270 [types all listed as deposited in ZMHB, but he was apparently referring to the types of the junior synonym, *Parastagmatoptera theresopolitana*Giglio-Tos 1914]; Otte and Spearman 2005: 294; Agudelo et al. 2007: 124; Lombardo et al. in press [Junior synonym of *Mantis flavoguttata* Audinet Serville, 1839].

#### Types:

**Figure 16.**
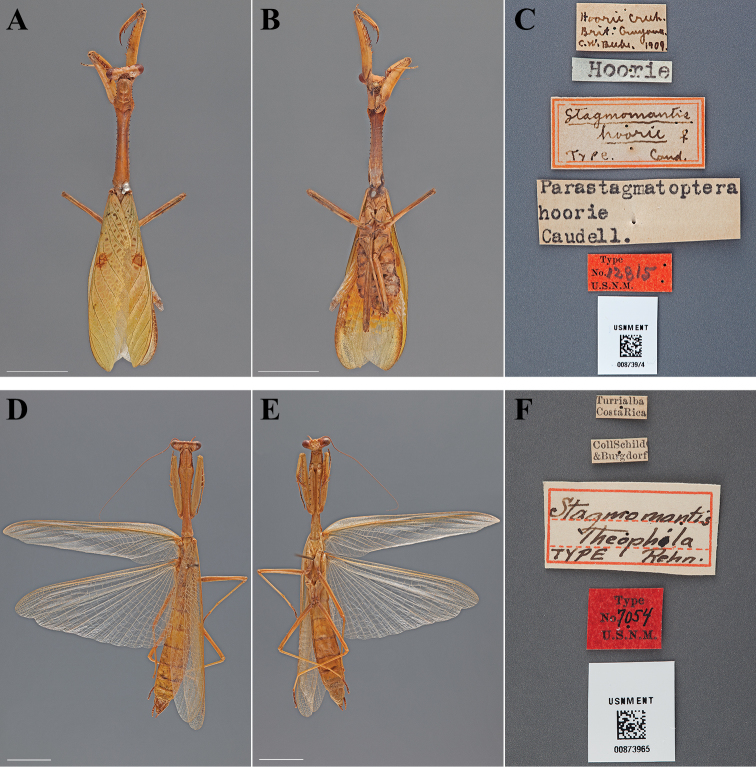
Types (scale bars = 1 cm). *Stagmomantis hoorie* Caudell, 1910 paralectotype female (USNM ENT 00873974): **A** dorsal habitus **B** ventral habitus **C** labels. *Stagmomantis theophila* Rehn, 1904 holotype male (USNM ENT 00873965): **D** dorsal habitus **E** ventral habitus **F** labels.

Lectotype Male (Fig. 15D–F; USNM ENT 00873973). Paralectotype Female (Fig. 16A–C; USNM ENT 00873974). One male and one female were described by Caudell (1910), neither being designated as the sole name-bearing type specimen. Therefore, they are syntypes under Article 72.1.1 of the Code. Herewithin, the male specimen is designated as the lectotype, the female the paralectotype under Article 74.1.1 of the Code. No prior fixation of a sole name-bearing type specimen was found in the literature.

#### Lectotype labels.

Hoorie Creek – Brit. Guyana. – C.W. Beebe, 1909. / Hoorie / Stagmomantis – hoorie ♂ / TYPE. Caud. / Type – No. 12815 – U.S.N.M.

#### Paralectotype labels.

Hoorie Creek – Brit. Guyana. – C.W. Beebe, 1909. / Hoorie / Stagmomantis – hoorie ♀ / TYPE. Caud. / Type – No. 12815 – U.S.N.M. / Parastamatoptera – hoorie – Caudell. [Cat. No. 12815, U.S.N.M.; referenced in the original description]

**Table:** 

Lectotype	Male	Guyana, Hoorie Creek	1909	7.538874, -59.418520	
Paralectotype	Female	Guyana, Hoorie Creek	1909	7.538874, -59.418520	

#### Measurements.

*Lectotype male*. Body length 44.73; forewing length 25.39; hindwing length 25.32; pronotum length 14.62; prozone length 2.78; pronotum width 2.24; pronotum narrow width 1.43; head width 4.81; head vertex to clypeus 1.88; frons width 1.63; frons height 0.66; prothoracic femur length 9.29; mesothoracic femur length 8.19; mesothoracic tibia length 6.61; mesothoracic tarsus length 3.91; metathoracic femur length 9.14; metathoracic tibia length 8.95; metathoracic tarsus length 5.94; discoidal femoral spines R4/L4; anteroventral femoral spine count R15/L15; posteroventral femoral spine count R4/L4; anteroventral tibial spine count R15/L15; posteroventral tibial spine count R13/L13.

*Paralectotype female*. Body length 49.69; forewing length 29.16; pronotum length 18.69; prozone length 3.68; pronotum width 3.25; pronotum narrow width 1.82; head width 6.04; head vertex to clypeus 2.52; frons width 2.05; frons height 1.00; prothoracic femur length 12.36; mesothoracic femur length 10.61; mesothoracic tibia length 8.39; mesothoracic tarsus length 4.41; metathoracic femur length 11.55; metathoracic tibia length 11.40; metathoracic tarsus length 6.75; discoidal femoral spines R4/L4; anteroventral femoral spine count R15/L15; posteroventral femoral spine count R4/L4; anteroventral tibial spine count R16/L16; posteroventral tibial spine count R14/L14.

### Stagmomantis theophila

Taxon classificationAnimaliaMantodeaMantidae

Rehn, 1904

Stagmomantis theophila : Rehn 1904: 563-564; Kirby 1904: 252; Hebard 1923: 340; Hebard 1924: 131; Hebard 1933a: 29; Hebard 1933b: 121; Beier 1935: 96; Terra 1995: 70; Salazar 1999: 11; Ehrmann 2002: 332 [Holotype listed as deposited in ANSP]; Agudelo 2004: 57; Otte and Spearman 2005: 212; Agudelo et al. 2007: 124.
Stauromantis theophila : Giglio-Tos 1917: 54; Giglio-Tos 1927: 384; Salazar 2002: 126.

#### Type.

Holotype Male (Fig. 16D–F; USNM ENT 00873965). The male specimen was referred to as the “Type” by Rehn (1904) and under Article 73.1.1 of the Code this sole name-bearing male specimen is the holotype.

#### Holotype labels.

Turrialba – Costa Rica / Coll Schild – & Burgdorf / Stagmomantis – theophila – TYPE Rehn. / Type – No. 7054 – U.S.N.M. [Cat. No. 6952, U.S.N.M.; referenced in the original description]

**Table:** 

Holotype	Male	Costa Rica, Turrialba	date unknown	9.904942, -83.688056	

#### Measurements.

Body length 64.42; forewing length 41.80; hindwing length 38.21; pronotum length 19.76; prozone length 4.40; pronotum width 3.56; pronotum narrow width 1.70; head width 6.81; head vertex to clypeus 2.32; frons width 2.24; frons height 0.92; prothoracic femur length 12.82; mesothoracic femur length 11.34; mesothoracic tibia length 8.66; mesothoracic tarsus length 6.18; metathoracic femur length 13.12; metathoracic tibia length 11.96; metathoracic tarsus length 8.10; discoidal femoral spines R4/L4; anteroventral femoral spine count R15/L15; posteroventral femoral spine count R4/L4; anteroventral tibial spine count R14/L14; posteroventral tibial spine count R11/L11.

### Tarachodes pilosipes

Taxon classificationAnimaliaMantodeaTarachodidae

Rehn, 1912

Tarachodes pilosipes : Rehn 1912: 453-455; Giglio-Tos 1927: 88 [Junior SYN of *Tarachodes insidiator* Wood-Mason, 1882]; Kaltenbach 1996: 311; Ehrmann 2002: 341 [SYN]; Otte and Spearman 2005: 355 [NON-SYN].

#### Type.

**Figure 17.**
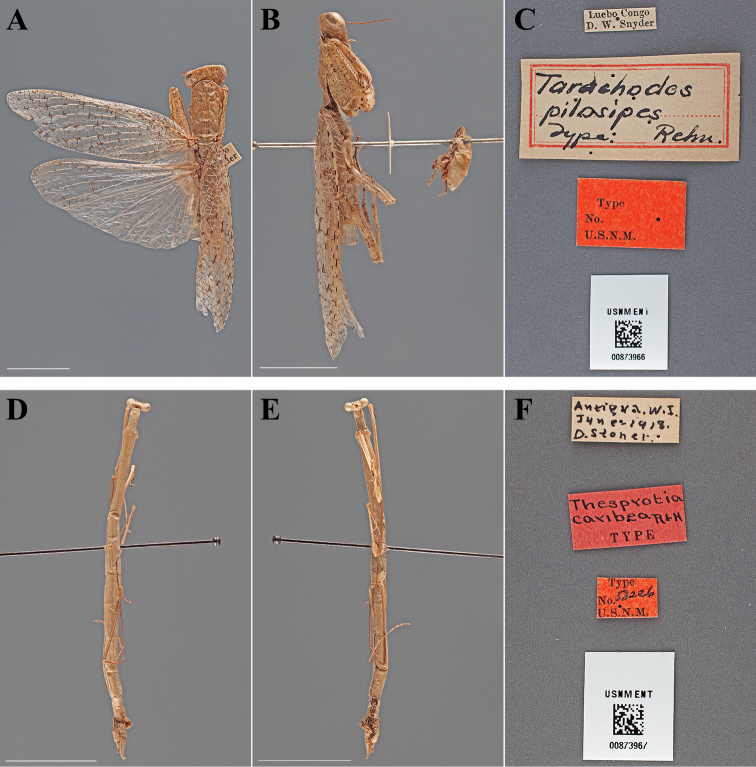
Types (scale bars = 1 cm). *Tarachodes pilosipes* Rehn, 1912 holotype male (USNM ENT 00873966): **A** dorsal habitus **B** ventral habitus **C** labels. *Thesprotia caribea* Rehn & Hebard, 1938 holotype female (USNM ENT 00873967): **D** dorsal habitus **E** ventral habitus **F** labels.

Holotype Male (Fig. 17A–C; USNM ENT 00873966). The male specimen was referred to as the “Type” by Rehn (1904) and under Article 73.1.1 of the Code this sole name-bearing male specimen is the holotype.

#### Holotype labels.

Luebo Congo – D. W. Snyder / Tarachodes – pilosipes – Type Rehn / Type – No. – U.S.N.M. [Cat. No. 14602, U.S.N.M.; referenced in the original description]

**Table:** 

Holotype	Male	Democratic Republic of Congo, Luebo	date unknown	-5.34980, 21.416844	

#### Measurements.

Body length 42.79; forewing length 31.60; hindwing length 26.62; pronotum length 9.60; prozone length 3.19; pronotum width 5.12; pronotum narrow width 4.28; head width 7.13; head vertex to clypeus 3.50; frons width 2.92; frons height 1.57; prothoracic femur length 8.86; mesothoracic femur length 6.32; mesothoracic tibia length 5.39; mesothoracic tarsus length 4.70; metathoracic femur length 7.35; metathoracic tibia length 7.05; metathoracic tarsus length 5.39; discoidal femoral spines R4/L4; anteroventral femoral spine count R13/L12; posteroventral femoral spine count R4; anteroventral tibial spine count R14/L14; posteroventral tibial spine count R15/L14.

### Thesprotia caribea

Taxon classificationAnimaliaMantodeaThespidae

Rehn & Hebard, 1938

Thesprotia caribea : Rehn and Hebard 1938: 36-38; Terra 1995: 50; Ehrmann 2002: 355 [Holotype and Allotype listed as deposited in ANSP]; Otte and Spearman 2005: 378; Agudelo et al. 2007: 118.

#### Types.

**Figure 18.**
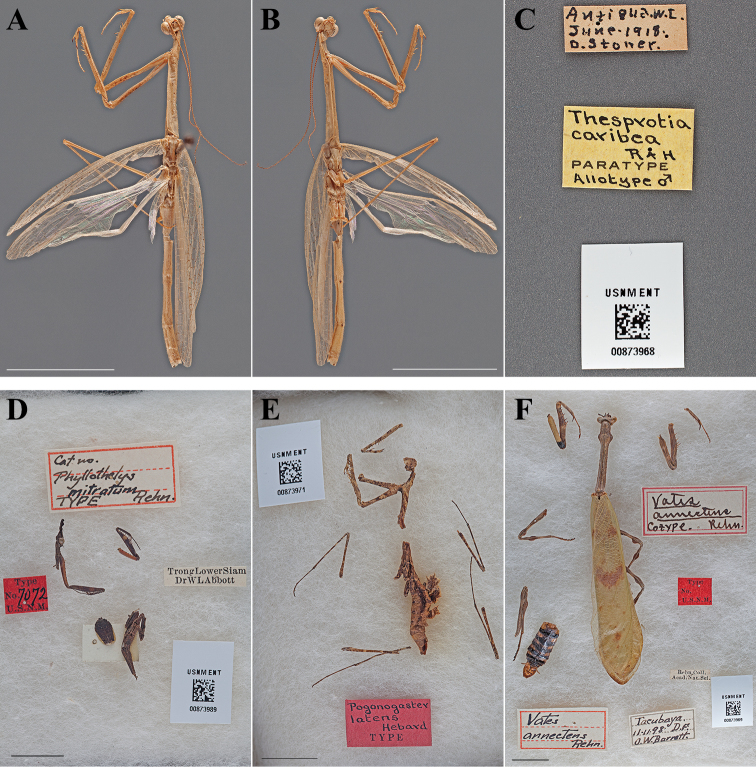
Types (scale bars = 1 cm). *Thesprotia caribea* Rehn & Hebard, 1938 allotype male (USNM ENT 00873968): **A** dorsal habitus **B** ventral habitus **C** labels. *Phyllothelys mitratum* Rehn, 1903 holotype female, immature (USNM ENT 00873989): **D** dorsal habitus and labels in Riker mount. *Pogonogaster latens* Hebard, 1919 holotype female (USNM ENT 00873971): **E** dorsal habitus and labels in Riker mount. *Vates annectens* Rehn, 1900 lectotype male (USNM ENT 00873969): **F** dorsal habitus and labels in Riker mount.

Holotype Female (Fig. 17D–F; USNM ENT 00873967). Allotype Male (Fig. 18A–C; USNM ENT 00873968). The female specimen was referred to as the “Type” by Rehn and Hebard (1938) and under Article 73.1.1 of the Code this sole name-bearing female specimen is the holotype.

#### Holotype labels.

Antigua. W.I. – June-1918 – D. Stoner / Thesprotia – caribea R+H – TYPE / Type – No. 53226 – U.S.N.M.

#### Allotype labels.

Antigua. W.I. – June-1918 – D. Stoner / Thesprotia – caribea – R+H – PARATYPE – Allotype ♂

**Table:** 

Holotype	Female	Antigua and Barbuda	Jun 1918	17.628958, -61.787811	
Allotype	Male	Antigua and Barbuda	Jun 1918	17.628958, -61.787811	

#### Measurements.

*Holotype female*. Body length 38.61; pronotum length 12.31; prozone length 3.00; pronotum width 1.71; pronotum narrow width 1.20; head width 2.66; head vertex to clypeus 1.35; frons width 0.86; frons height 0.30; prothoracic femur length 7.96; mesothoracic femur length 8.08; mesothoracic tibia length 7.92; mesothoracic tarsus length 3.72; metathoracic femur length 8.78; metathoracic tibia length 10.17; metathoracic tarsus length 4.59; discoidal femoral spines R3/L3; anteroventral femoral spine count R6/L6; posteroventral femoral spine count R1/L1; anteroventral tibial spine count R1/L1; posteroventral tibial spine count R1/L1.

*Allotype male*. Body length 32.33; forewing length 17.58; hindwing length 17.84; pronotum length 10.33; prozone length 2.74; pronotum width 1.34; pronotum narrow width 1.02; head width 2.41; head vertex to clypeus 1.23; frons width 0.80; frons height 0.26; prothoracic femur length 7.40; mesothoracic femur length 9.22; mesothoracic tibia length 9.03; mesothoracic tarsus length 4.94; discoidal femoral spines R3/L3; anteroventral femoral spine count R5/L6; posteroventral femoral spine count R1/L1; anteroventral tibial spine count R1/L1; posteroventral tibial spine count R1/L1.

### Vates annectens

Taxon classificationAnimaliaMantodeaMantidae

Rehn, 1900

Vates annectens : Rehn 1900: 85-86; Kirby 1904: 306; Rehn 1911: 18.
Pseudovates annectens : Giglio-Tos 1914: 48; Giglio-Tos 1927: 609; Hebard 1932: 213 [Junior synonym of *Theoclytes tolteca* Saussure, 1859]; Otte 1978: 77; Ehrmann 2002: 299 [SYN]; Otte and Spearman 2005: 314 [SYN]; Agudelo et al. 2007: 154 [SYN].

#### Type.

Lectotype Male (Fig. 18F; USNM ENT 00873969). One male and one female were described by Rehn (1900), neither being designated as the sole name-bearing type specimen. Therefore, they are syntypes under Article 72.1.1 of the Code. Herewithin, the male specimen is designated as the lectotype under Article 74.1.1 of the Code. No prior fixation of a sole name-bearing type specimen was found in the literature. The two specimens were listed as deposited in the “...private collection of the writer”, the male now at the USNM and the female likely in the ANSP according to Otte (1978).

#### Lectotype labels.

Tacubaya – 11.11.98 D.F. – O.W. Barrett. / Rehn Coll. – Acad. Nat. Sci. / Vates – annectens – Cotype. Rehn / Type – No. – U.S.N.M.

**Table:** 

Lectotype	Male	Mexico, Tacubaya	11 Nov 1898	19.400949, -99.18668	

#### Measurements.

Body length 70.91; forewing length 48.13; pronotum length 18.87; prozone length 3.42; pronotum width 4.04; pronotum narrow width 2.03; head width 5.41; frons width 2.09; frons height 1.01; prothoracic femur length 11.09; mesothoracic femur length 10.16; mesothoracic tibia length 8.83; mesothoracic tarsus length 6.55; metathoracic femur length 12.17; metathoracic tibia length 11.91; metathoracic tarsus length 8.27; discoidal femoral spines R4/L4; anteroventral femoral spine count R14/L13; posteroventral femoral spine count R4/L4; anteroventral tibial spine count R14/L13; posteroventral tibial spine count R10/L10.

### Additional paratype material

#### *Amantis basilana* Hebard, 1920

*Amantis basilana* Hebard 1920a: 34; Giglio-Tos 1927: 171; Beier 1935: 28; Otte 1978: 75; Ehrmann 2002: 56; Otte and Spearman 2005: 141.

**Table:** 

Paratype	Male	Island of Basilan, Baker, Amantis basilana, female Hebard, Paratype, Hebard Cln., USNM ENT00873053	date unknown	6.594951, 122.048546	

#### *Liturgusa tessae* Svenson, 2014

*Liturgusa tessae* Svenson 2014: 90.

**Table:** 

Paratype	Male	Brazil, Para: Rio Xingu, Camp (52°22'W, 3°39'S), ca 60 km S. Altamira, 8–12 Oct 1986, P. Spangler & O. Flint; USNM ENT00873005	8–12 Oct 1986	-3.650000, -52.366667	
Paratype	Male	Brazil, Para: Rio Xingu, Camp (52°22'W, 3°39'S), ca 60 km S. Altamira, 8-12 Oct 1986, P. Spangler & O. Flint; USNM ENT00873006	8-12 Oct 1986	-3.650000, -52.366667	
Paratype	Male	Peru, Rio Tambopata Res., 30 km (air) SW Pto. Maldonado, 290 m, 12°50'S, 69°17'W; Smithsonian Institution Canopy Fogging Project, T.L. Erwin et al., colls. 07 May 89, 05/02/051; USNM ENT00873048	07 May 1989	-12.833333, -69.283333	
Paratype	Female	Peru, Madre de Dios, Rio Tambopata Res., 30 km (air) SW Pto. Maldonado, 290 m, 12°50'S, 69°17'W; Smithsonian Institution Canopy Fogging Project, T.L. Erwin et al., colls. 08 Nov 83 May 89, 04/01/072; USNM ENT00873049	08 Nov 1983	-12.833333, -69.283333	

#### *Liturgusa trinidadensis* Svenson, 2014

*Liturgusa trinidadensis* Svenson 2014: 95.

**Table:** 

Paratype	Male	Trinidad, Arima Valley, B.W.I. 20-II-1952, Tropical Research Station, New York Zool Society; USNM ENT00873007	20 Feb 1952	10.661851, -61.289723	
Paratype	Female	Trinidad, Arima Valley, B.W.I. 8-II-1952, Tropical Research Station, New York Zool Society; USNM ENT00873008	11 Aug 1952	10.661851, -61.289723	
Paratype	Male	Trinidad, Jun WI, Aug. Busck Collector; USNM ENT00873009	date unknown	?	?
Paratype	Male	Trinidad, Jun WI, Aug. Busck Collector; USNM ENT00873015	date unknown	?	?
Paratype	Male	Trinidad, Aug-22-1907, O.W. Barria, On Cacao, 252; USNM ENT00873016	22 Aug 1907	?	?

#### *Sinomiopteryx grahami* Tinkham, 1937

*Sinomiopteryx grahami* Tinkham 1937: 495; Otte 1978: 77; Ehrmann 2002: 319; Otte and Spearman 2005: 383.

**Table:** 

Paratype	Male	China, Szechuan, DC Graham Coll, Paratype No. 52047 U.S.N.M., *Sinomiopteryx grahami*, 1937 Tinkham, Paratype male, USNM ENT 00873054	date unknown	29.581773, 103.290837	
Paratype	Male	China, Mt Omei - 1923, Szechuan China, DC Graham Collector, Paratype No. 52047 U.S.N.M., *Sinomiopteryx grahami*, 1937 Tinkham, Paratype male, USNM ENT 00873055	1923	29.581773, 103.290837	
Paratype	Male	China, Shin Kai Si, Mt Omei, Szechuan China, Aug 6-15-1921, DC Graham Collector, Paratype No. 52047 U.S.N.M., *Sinomiopteryx grahami*, 1937 Tinkham, Paratype male, USNM ENT 00873056	6–15 Aug 1921	29.581773, 103.290837	
Paratype	Male	China, Suifu, Szechuan China, Aug 15-30 1921, DC Graham Collector, Paratype No. 52047 U.S.N.M., *Sinomiopteryx grahami*, 1937 Tinkham, Paratype male, USNM ENT 00873057	15–30 Aug 1921	28.628051, 104.413940	
Paratype	Male	China, Shin Kai Si, Mt Omei, Szechuan China, 8-19-26, DC Graham Collector, Altitude 4500 ft, Paratype No. 52047 U.S.N.M., *Sinomiopteryx grahami*, 1937 Tinkham, Paratype male, USNM ENT 00873058	19 Aug 1926	29.581773, 103.290837	

#### *Stagmomantis floridensis* Davis, 1919

*Stagmomantis floridensis* Davis 1919: 4; Beier 1935: 95; Terra 1995: 70; Ehrmann 2002: 331; Otte and Spearman 2005: 209.

**Table:** 

Paratype	Female	United States of America, Parish Fla., x.20.1916, Wm. T. Davis Collection, Stagmomantis floridensis Paratype, 3., USNM ENT00873052	20 Oct 1916	27.586813, -82.424193	

### Mistaken type material

#### *Cruentosaga phanatica* Rehn

An immature female specimen was located in the type collection bearing the following label data: Trong Lower Siam – Dr WL Abbott / Cat.no. – Cruentosaga – phanatica – Type Rehn / Type No. 7070 USNM / = *Theopropus elegans*. The name, *Cruentosaga phanatica*, was not located in the literature. The type status was therefore doubted and a search in publications by James Rehn for potential reference to this specimen revealed a potential mistake. In a paper focused on “Old World” Mantodea (Rehn 1903: 717), a specimen identified as *Theopropus elegans* Westwood, 1832, matches the label data as well as the physical description. A reference to the type for the genus *Theopropus* immediately preceded the presentation of this specimen, which may have caused confusion about the specimen’s type status. Although the origin of the name *Cruentosaga phanatica* is not known, it is strongly believed that USNM Type No. 7070 is not a name bearing specimen but merely a determined specimen by Rehn within a publication surveying Old World Mantodea.

#### Mantis caldwellii

Taxon classificationAnimaliaMantodeaMantidae

Bates, 1863

Mantis caldwellii : Bates 1863: 479.
Popa caldwellii : Saussure 1871: 309.
Hierodula caldwellii : Saussure and Zehntner 1895: 192.
Tarachomantis caldwellii : Kirby 1904: 241; Giglio-Tos 1907: 3; Giglio-Tos 1912: 27; Giglio-Tos 1913: 405; Giglio-Tos 1927: 424; Beier 1935: 80; Ehrmann 2002: 347; Otte and Spearman 2005: 277.

##### Remarks.

Two females were mentioned by Bates (1863) after the description that was based on females, but neither was designated as the sole name-bearing type specimen. Neither of the two mentioned specimens were located in the USNM collection or are listed in the records of the museum. The species was listed as deposited in the USNM collection by Ehrmann (2002) and Otte and Spearman (2005).

## Supplementary Material

XML Treatment for Acromantis palauana

XML Treatment for Ameles malaccana

XML Treatment for Amorphoscelis chinensis

XML Treatment for Amorphoscelis pantherina

XML Treatment for Calidomantis hosia

XML Treatment for Danuria angolensis

XML Treatment for Galapagia solitaria

XML Treatment for Galepsus congicus

XML Treatment for Gonatista major

XML Treatment for Harpagonyx carlottae

XML Treatment for Hoplocorypha boviformis

XML Treatment for Hoplocorypha perplexa

XML Treatment for Liturgusa dominica

XML Treatment for Liturgusa manausensis

XML Treatment for Liturgusa neblina

XML Treatment for Liturgusa zoae

XML Treatment for Mantis viridis

XML Treatment for Oxyopsis oculea

XML Treatment for Palaeothespis oreophilus

XML Treatment for Panurgica fratercula

XML Treatment for Panurgica liberiana

XML Treatment for Phyllothelys mitratum

XML Treatment for Popa batesi

XML Treatment for Pogonogaster latens

XML Treatment for Rhombodera megaera

XML Treatment for Stagmatoptera insatiabilis

XML Treatment for Stagmomantis hoorie

XML Treatment for Stagmomantis theophila

XML Treatment for Tarachodes pilosipes

XML Treatment for Thesprotia caribea

XML Treatment for Vates annectens

XML Treatment for Mantis caldwellii
